# Strategies for Glycoengineering Therapeutic Proteins

**DOI:** 10.3389/fchem.2022.863118

**Published:** 2022-04-13

**Authors:** Kris Dammen-Brower, Paige Epler, Stanley Zhu, Zachary J. Bernstein, Paul R. Stabach, Demetrios T. Braddock, Jamie B. Spangler, Kevin J. Yarema

**Affiliations:** ^1^ Translational Tissue Engineering Center, Johns Hopkins School of Medicine, Baltimore, MD, United States; ^2^ Department of Biomedical Engineering, The Johns Hopkins University, Baltimore, MD, United States; ^3^ Department of Pathology, Yale University School of Medicine, New Haven, CT, United States; ^4^ Department of Chemical and Biomolecular Engineering, The Johns Hopkins University, Baltimore, MD, United States; ^5^ Department of Oncology, Johns Hopkins School of Medicine, Baltimore, MD, United States; ^6^ Bloomberg-Kimmel Institute for Cancer Immunotherapy, Sidney Kimmel Comprehensive Cancer Center, Johns Hopkins School of Medicine, Baltimore, MD, United States; ^7^ Department of Ophthalmology, Wilmer Eye Institute, Johns Hopkins School of Medicine, Baltimore, MD, United States; ^8^ Department of Molecular Microbiology and Immunology, Johns Hopkins Bloomberg School of Public Health, Baltimore, MD, United States

**Keywords:** glycoengineering, pharmacodynamics, pharmacokinetics, therapeutic, glycosylation, N-glycans, biomanufacturing

## Abstract

Almost all therapeutic proteins are glycosylated, with the carbohydrate component playing a long-established, substantial role in the safety and pharmacokinetic properties of this dominant category of drugs. In the past few years and moving forward, glycosylation is increasingly being implicated in the pharmacodynamics and therapeutic efficacy of therapeutic proteins. This article provides illustrative examples of drugs that have already been improved through glycoengineering including cytokines exemplified by erythropoietin (EPO), enzymes (ectonucleotide pyrophosphatase 1, ENPP1), and IgG antibodies (e.g., afucosylated Gazyva^®^, Poteligeo^®^, Fasenra™, and Uplizna^®^). In the future, the deliberate modification of therapeutic protein glycosylation will become more prevalent as glycoengineering strategies, including sophisticated computer-aided tools for “building in” glycans sites, acceptance of a broad range of production systems with various glycosylation capabilities, and supplementation methods for introducing non-natural metabolites into glycosylation pathways further develop and become more accessible.

## 1 Introduction

This report describes the impact of glycosylation on the pharmacokinetics, pharmacodynamics, therapeutic activity, and production (biomanufacturing) of therapeutic proteins using several examples that illustrate strategies and methods to glycoengineer this important class of drugs for increased effectiveness. In Section 2, we describe how glycosylation affects the pharmacokinetics (PK) of protein-based drugs; defined simply, PK is the study of the effects of the body on a drug including absorption, distribution, metabolism, and excretion. Next, in Section 3 and Section 4, we describe how glycosylation modulates a drug’s pharmacodynamic (PD) properties, which are defined as the effects of the drug on the body and the body’s biochemical and physiological responses to a drug. More specifically, Section 3 covers several classes of therapeutic proteins whose PD activities depend on glycosylation, including enzymes, hormones, and blood-acting factors. Section 4 covers therapeutic antibodies, which constitute the largest class of protein-based drugs and have unique glycosylation features compared to most proteins. Finally, in Section 5 we provide an overview of methods for controlling and modulating this glycosylation during the design and biomanufacturing of therapeutic proteins. Throughout each section we provide illustrative examples of therapeutic proteins but emphasize that our examples are not complete or exhaustive.

**FIGURE 1:**
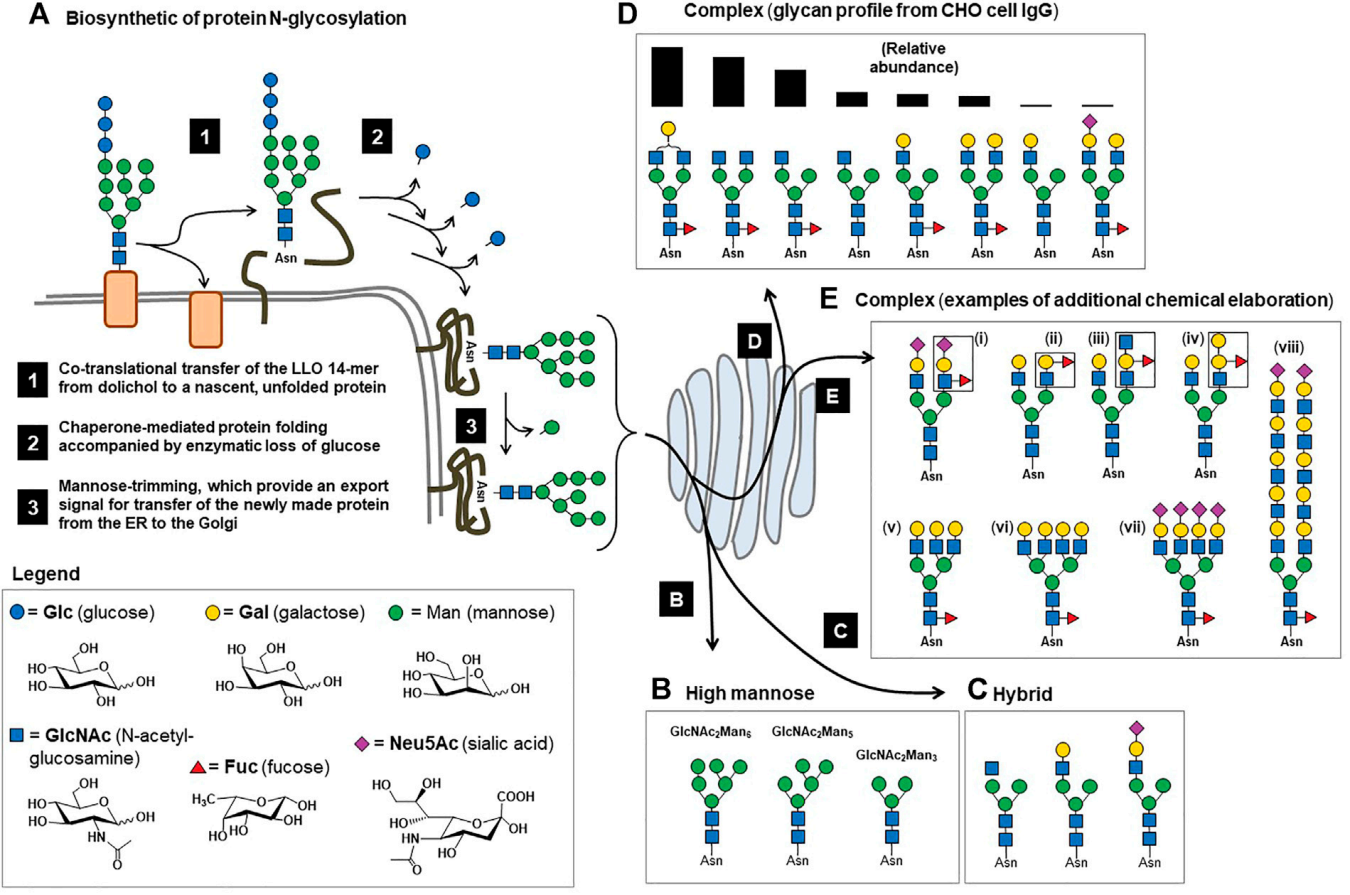
Overview of mammalian N-glycosylation. **(A)** Step 1. The LLO 14-mer structure shown (GlcNAc_2_Man_9_Glc_3_) is co-translationally transferred from dolichol phosphate to an asparagine residue of a nascent unfolded protein by oligosaccharyltransferase (OST) in the ER (Breitling & Aebi 2013). Step 2. Chaperone-mediated protein folding occurs concomitant with glucose trimming, generating a (in Step 3) a GlcNAc_2_Man_9_ or GlcNAc_2_Man_8_ structure that functions as an export signal for the transfer of successfully folded proteins to the Golgi (Helenius & Aebi 2001). **(B)** In the Golgi, further trimming of mannose residues occurs to produce a series of GlcNAc_2_Man_n_ structures referred to as “high mannose”-type N-glycans, where n is typically between 3 and 6. **(C)** Also in the Golgi, one, two, or three GlcNAc residues are added to a GlcNAc_2_Man_n_ structures, which can be further elaborated (e.g., with galactose and sialic acid, as shown) producing “hybrid” type N-glycans when a single GlcNAc is added to a GlcNAc_2_Man_n_ structure. Hybrid N-glycans typically are low in abundance and have few known roles in therapeutic proteins. A larger proportion of N-glycans have GlcNAc residues added to both terminal mannose residues of the GlcNAc_2_Man_3_ structure, most frequently resulting in small biantennary structures (Werz et al., 2007) such as those shown in Panel **(D)**, where the glycoprofile of IgG Fc domain N-glycans from one study (del Val et al., 2016) are shown rank ordered by their relative abundance. **(E)** A relatively small proportion (generally 5% or less) of N-glycans are further elaborated, resulting in epitopes such as **(i)** sialyl Lewis x (sLe^x^), the H, A, and B blood type antigens **[(ii), (iii)**, and **(iv)**, respectively**]**; **(v)** tri- and **(vi)** tetra-antennary structures that can be unsialylated to fully sialylated **(vii)**; and finally certain N-glycans have extended “LacNAc” repeats (four are shown) that can serve as preferred ligands for certain receptors, such as the hemagglutinin protein of the influenza virus (Ji et al., 2017), whereas glycans from EPO can have single LacNAc repeats (Cowper et al., 2018).

Before covering these topics in detail, here in the Introduction (Section 1), we briefly describe key concepts related to the glycosylation of therapeutic proteins (Figure 1). With few exceptions (e.g., regulatory peptides and small hormones such as insulin), all therapeutic proteins have at least one, and often several, N-glycans. Overall, approximately 50% of human proteins are glycosylated, which governs their folding, intracellular and extracellular trafficking, stability, circulatory half-life, and immunogenicity (Olden et al., 1982; Breitfeld et al., 1984; Dwek 1996; Willey 1999; Dwek & Butters 2002). Mammalian glycosylation is remarkably complex, consisting of N-linked glycans, O-linked glycans, C-linked glycans, phosphoglycosylation, and glypiation. In this article, we will almost exclusively discuss N-linked glycosylation, because clinical translational glycoengineering efforts have overwhelmingly focused on this type of glycosylation to date.

From a biochemical perspective, virtually all cell surface or secreted proteins (i.e., candidates for drug development) are N-glycosylated, which occurs co-translationally when the lipid-linked oligosaccharide (LLO) GlcNAc_2_Man_9_Glc_3_ 14-mer structure is added to a consensus sequon (Figure 1A, Step 1). This structure is critical for chaperone-mediated protein folding in the endoplasmic reticulum (ER) (Helenius & Aebi 2001), where the three glucose residues are sequentially trimmed during the folding process (Figure 1A, Step 2). Successfully folded proteins with a GlcNAc_2_Man_9_, or a slightly trimmed GlcNAc_2_Man_8_ structure (Figure 1A, Step 3), are exported to the Golgi where mannosidases trim additional mannose residues, ultimately resulting in GlcNAc_2_Man_5_ to GlcNAcMan_3_ structures (Figure 1B). In some cases, these “high mannose” glycans appear on mature proteins without further processing and affect the proteins’ distribution and by extension, their bioactivities. In other cases, the resulting GlcNAc_2_Man_n_ glycans are precursor structures for further elaboration in the Golgi, forming hybrid (Figure 1C) and complex type N-glycans. In most cases, the ultimate complex type N-glycans are relatively small in size; for perspective, ∼90% of mammalian glycans are comprised of 12 or fewer monosaccharides (Werz et al., 2007), which covers the size range for Fc-domain glycans of IgG antibodies (Figure 1D). Less frequently, complex type N-glycans can be considerably larger (Figure 1E), as found on therapeutic proteins such as erythropoietin (EPO).

## 2 Pharmacokinetics

Historically, the effects of glycosylation on therapeutic proteins were first evident through changes to their pharmacokinetic (PK) properties (Liu 2015; Liu 2018; Boune et al., 2020). Accordingly, we begin by describing the impact of glycosylation on the PK of protein drugs. Definitions of PK include “the movement of drugs through the body” or “the study of what the body does to a drug,” and includes a drug’s absorption, distribution, metabolism, and excretion; this set of metrics is typically abbreviated “ADME” (Tibbitts et al., 2016).

### 2.1 Serum Clearance

One of the earliest contexts where glycosylation was recognized to be important for therapeutic proteins was through serum clearance. This endpoint was evident from studies with erythropoietin (EPO), a drug that pioneered the importance of glycoengineering for improving biologics. Specifically, glycoengineering improved the PK properties of EPO by modulating two ways that glycans contribute to serum clearance, and ultimately, drug elimination. These mechanisms are kidney filtration, which can be slowed by increasing the size of a protein by adding N-glycan sites (Section 2.1.1) and avoiding receptor-mediated clearance by the asialoglycoprotein receptor [(ASPGR) Section 2.1.2] or the mannose receptor [(MR) Section 2.1.3].

#### 2.1.1 N-Glycans Add Steric Bulk and Increase Hydrodynamic Radius to Avoid Kidney Filtration

The efficiency of kidney filtration rapidly increases as protein’s size falls below ∼40 kDa; for example, the glomerular sieving coefficient of the anionic form of horseradish peroxidase (40 kDa) is 0.007, compared to 0.33 for superoxide dismutase (∼32 kDa) and 0.75 for myoglobin (16.9 kDa) (Maack et al., 1979; Tsao et al., 1991). Erythropoietin has a molecular weight of ∼18.4 kDa based on its amino acid sequence, suggesting that it should experience kidney filtration similar to myoglobin. Although wild-type EPO is cleared from the serum relatively rapidly [its half-life ranges between 5 and 11 h (Elliott et al., 2008)], EPO produced with truncated N-glycans had substantially (∼7-fold) faster clearance (Wasley et al., 1991). The glycosylation of EPO has now been thoroughly characterized, with the protein’s three N-glycans contributing ∼12 kDa of the glycoprotein’s total mass of ∼30.4 kDa; each N-glycan is typically a tri- or tetra-antennary structure that is highly sialylated and often has LacNAc repeats (Figure 1E). These large glycans are particularly effective at avoiding glomerular filtration, because unlike amino acid chains that fold into compact proteins, they are fully extended in the aqueous physiological milieu; furthermore, they are motile, allowing them to “sweep out” space.

**FIGURE 2:**
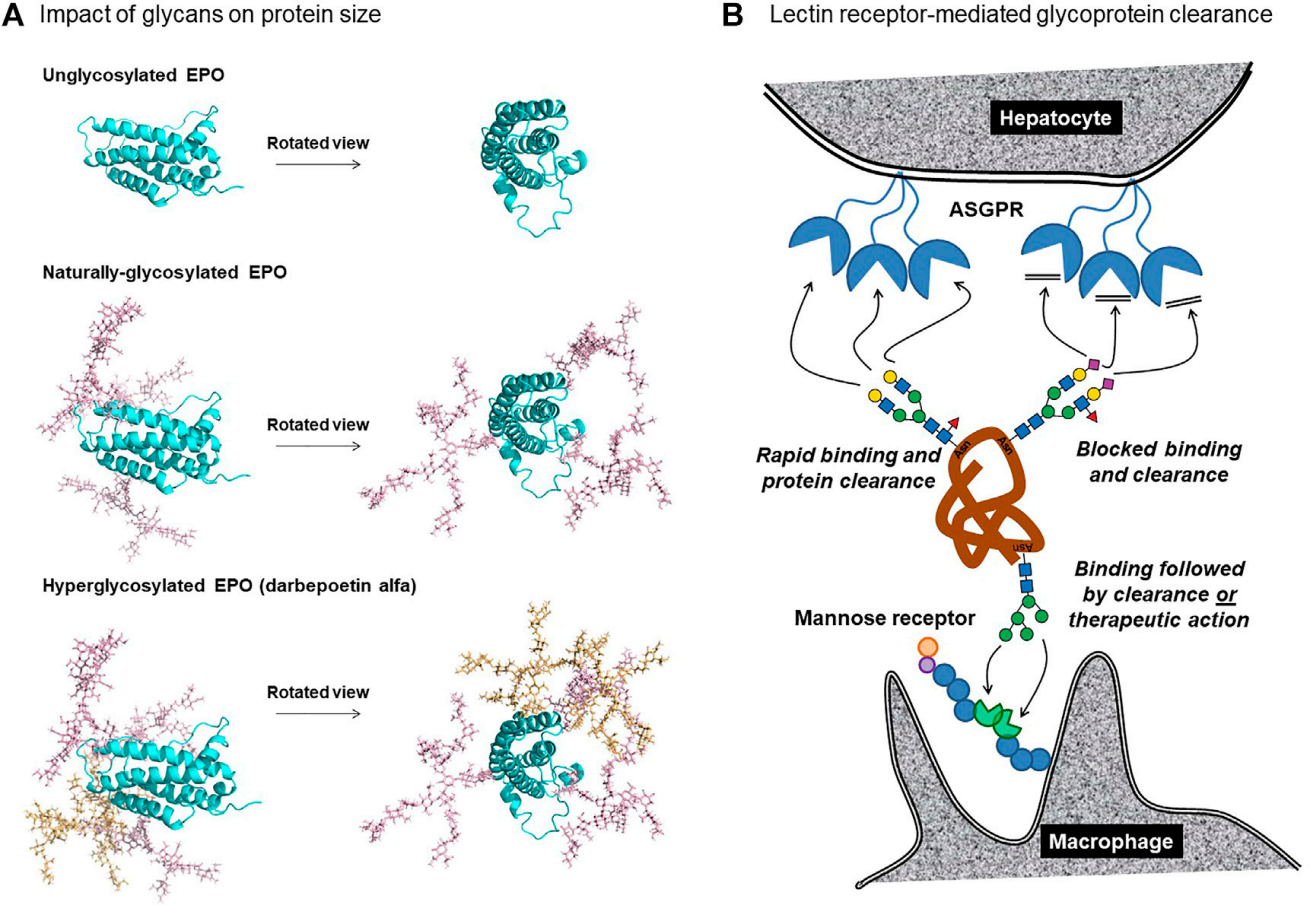
N-glycans influence the clearance of therapeutic proteins based (A) on size and (B) receptor-mediated clearance. **(A)** Unglycosylated EPO (top) is compared with naturally glycosylated EPO, which has three N-glycans at Asn24, N38, and N83 (middle) and with darbepoetin alfa, which has five glycans including those newly-added at Asn30 and N88 (bottom). The glycan structures depicted are representative of the experimentally-determined N-glycan profile of EPO (Cowper et al., 2018), in particular the structure shown in Figure 1E(vii). Protein models were generated using SWSS-MODEL software (Waterhouse et al., 2018) and modified to present N-glycan structures via CHARMM (Jo et al., 2008) and PyMOL (PyMOL Molecular Graphics System, Version 2.0, Schrödinger, LLC). The darbepoetin alfa sequence was obtained from the KEGG Drug database. **(B)** Lectin receptor-mediated clearance removes proteins from circulation through ASPGR binding of galactose-terminated glycans (top left); addition of sialic acid masks the galactose blocking binding and clearance (top right). Mannose-terminated glycans bind to mannose receptors on macrophages, dendritic cells, dermal fibroblasts, and keratinocytes, resulting in clearance or, in some cases, therapeutic activity [for example to treat Gaucher disease (Section 2.3.3)].

These two factors enable glycans to increase the hydrodynamic radius of a protein more effectively than a commensurate increase in peptide mass; for example, RNAse is a ∼15 kDa protein whose hydrodynamic radius is doubled through attachment of a small, biantennary N-glycan of ∼2 kDa (Dwek 1996). Similarly, the size of glycosylated EPO is dramatically larger than non-glycosylated EPO (Figure 2A). Although the larger size of naturally-glycosylated EPO improves its serum longevity by ∼7-fold compared to aglycosylated protein (Wasley et al., 1991), its molecular weight of ∼30.4 kDa suggested that further improvements were possible because proteins greater than ∼40 kDa have even lower glomerular sieving coefficients; for example, the coefficient for superoxide dismutase [32 kDa] of 0.33 is reduced to 0.007 for horseradish peroxidase [40 kDa]. Accordingly, the addition of two N-glycans to EPO to form hyper-glycoengineered darbepoetin alfa (Aranesp^®^) (Figure 2A) increased the drug’s molecular weight to ∼37–38 kDa, slowing serum clearance from ∼8 to ∼25 h (Macdougal 2002; Egrie et al., 2003; Elliott et al., 2008).

A limitation to glycoengineering strategies designed to avoid glomerular sieving and concomitant kidney filtration is that they depend on the target protein being appropriately sized. On one hand, if a protein or peptide is too small (e.g., insulin and/or interleukins), it may not be possible to add a sufficient number of N-glycans to enlarge the protein above the ∼40 kD size threshold without loss of biological activity. In particular, EPO illustrates how both the natural and glycoengineered glycans are oriented towards one side of the protein (Figure 2A). In retrospect, this orientation was critical to avoid steric interference with its binding to its partner proteins; similarly fortuitous submolecular siting of built-in glycans may not be possible for all therapeutic proteins. In other cases, [e.g., ENPP-1 (Section 2.2.1) and therapeutic antibodies (Section 4)], the proteins are already above the threshold for kidney filtration, and any further increase in steric bulk is unlikely to provide additional improvement in serum longevity. In other words, EPO was ideally situated for glycoengineering due to its size, which was marginally below the threshold where glomerular sieving becomes ineffective. Nevertheless, the “size matters” principle is likely to benefit at least some additional therapeutic proteins. For example, efforts are underway to produce glycoengineered insulin (Guan et al., 2018) and glucagon (Higashiyama et al., 2018; Ichikawa et al., 2018); addition of glycans will substantially increase the hydrodynamic radius of these small proteins, potentially slowing kidney filtration.

#### 2.1.2 Sialylation Masks Asialoglycoprotein Receptor-Mediated Clearance

As just discussed, adding steric bulk to a therapeutic protein *via* glycosylation can be an effective albeit limited strategy to improve PK properties. A more general glycan-related clearance mechanism involves receptor-mediated cellular uptake by lectin receptors. The dominant example of this mechanism involves hepatic clearance of serum proteins *via* the asialoglycoprotein receptor [ASGPR (Ashwell & Harford 1982)]. The ASPGR functions by multivalent recognition of the terminal galactose residues of non-sialylated N-glycans, rapidly depleting the host proteins from circulation (Schwartz 1984; Weigel 1994) (Figure 2B). The effectiveness of this mechanism for removing “aged” proteins from the serum as they lose their terminal sialic acids over time, thereby exposing their otherwise penultimate galactose moieties, is illustrated by deliberately desialylated EPO, which has a serum half-life of ∼10 min. By contrast, normally sialylated EPO has a serum half-life ranging from 5 to 11 h (Elliott et al., 2008). The increased serum longevity of darbepoetin alfa is not only attributed to increased size (Figure 2A) but also to hypersialylation, having as many as 22 copies of sialic acid (Elliot et al., 2000), which helps it avoid ASGPR clearance (Figure 2B). This pioneering example illustrates the general importance of high sialic site acid occupancy for prolonged *in vivo* circulation of therapeutic proteins. As an aside, sialic acid can improve the safety of therapeutic proteins by a similar masking mechanism where this sugar obscures underlying antigenic epitopes, reducing the generation of neutralizing antibodies (Bork et al., 2009; Li & d'Aniou 2009).

#### 2.1.3 Mannose Receptor-Mediated Glycoprotein Clearance

Glycoproteins also can be recognized by mannose-binding receptors (MRs) on various cell types, including hepatocytes, fibroblasts, and endothelial cells, as well as by immune cells such as macrophages and dendritic cells (Schlesinger et al., 1978; Sheikh et al., 2000). These receptors have multiple functions. One function is to rapidly clear proteins with high mannose-type glycans (Figure 1B), as well as GlcNAc and fucose-containing glycans (Feinberg et al., 2021), such as the glycoprotein hormone lutropin. In general, these receptors help maintain serum glycoprotein homeostasis (Roseman & Baenziger 2000; Lee et al., 2002). A second function of MRs is to facilitate the phagocytosis of pathogens such as *Candida albicans*, *Pneumocystis carnini,* and *Leishmania donovan* whose surfaces are covered with mannose-terminated glycans. These glycans allow the removal of these pathogens from the host by macrophages as well as by non-immune cells that also express mannose receptors such as keratinocytes (Szolnoky et al., 2001; Gazi & Martinez-Pomares 2009). A third and also immunomodulatory function of MRs is to enhance soluble, but not cell-associated antigens, for cross-presentation (Burgdorf et al., 2006).

Another aspect of human immune response to pathogens is the generation of inflammatory glycoproteins such as hydrolases, tissue plasminogen activator, and myeloperoxidase, which can be damaging to host tissues if retained after the infection has been resolved; high-mannose glycans on these glycoproteins provide these conditionally protective factors with quick clearance *via* cells with MRs, helping to avoid post-infection damage to the host (Lee et al., 2002; Gazi & Martinez-Pomares 2009). From a drug development standpoint, the ability of certain cells to internalize mannose-terminated glycans has been exploited to direct therapeutic proteins to cell types such as macrophages, as described for Gaucher’s disease in Section 2.3.3.

#### 2.1.4 IgG Antibodies: An Exception to Rapid Clearance

Therapeutic antibodies, which to date are almost all IgGs, are outliers compared to other therapeutic proteins because they are not subject to the two “universal” clearance mechanisms just discussed (size-based kidney filtration and glycan-based receptor clearance). First, IgG antibodies are large (∼150 kD), well above the size range susceptible for kidney filtration. Second, the N-glycans of commercial IgG antibodies are uniquely oriented inwards, being “buried” between the two Fc region protein domains, making them largely inaccessible to ASGPR clearance despite their low sialylation status (Figure 1D). In addition to glycan-based clearance mechanisms, the Fc domain of IgG antibodies binds to the neonatal Fc receptor, which directs intracellular trafficking to avoid proteosomal degradation upon uptake into the cell by re-releasing the antibody into circulation. These factors provide therapeutic antibodies with *in vivo* half-lives ranging from several days to many weeks (Ryman & Meibohm 2017; Liu 2018; Ovacik & LIn 2018) instead of the several hours typical of most other protein-based drugs. For example, the half-life of the commercial anti-HER2 antibody drug trastuzumab is 28 days (Boekhout et al., 2011), even though only ∼1.1% of its Fc N-glycans are sialylated (Nakano et al., 2009).

### 2.2 Absorption and Distribution

Unlike the well-known role for glycosylation in the elimination of therapeutic proteins and in already-approved glycoengineered drugs such as darbepoetin alfa that exploit glycans for improved circulatory half-life, the role of glycoengineering in modulating the absorption and distribution of these drugs throughout the body is in relative infancy. Nevertheless, two case studies (ENPP1-Fc, Section 2.2.1 and hyaluronidase, Section 2.2.2) demonstrate the intriguing potential for exploiting glycoengineering to improve the absorption and biodistribution of therapeutic proteins. In this discussion, we focus on subcutaneous delivery. Subcutaneously injected therapeutics have been popular for their potential convenience for physicians, patients at greater risk for systemic reactions, and those in which constant venous access is difficult to maintain (particularly infants) (Turner & Balu-Iyer 2018). Furthermore, subcutaneous delivery often allows patient self-administration, reducing the cost, stress, and inconvenience of repeated administration at a healthcare center. These benefits have made subcutaneous administration appealing to a growing number of therapeutic proteins, including cytokines, human insulin, and immunoglobulins (Turner & Balu-Iyer 2018).

#### 2.2.1 Absorption of Glycoengineered ENPP-1

**FIGURE 3:**
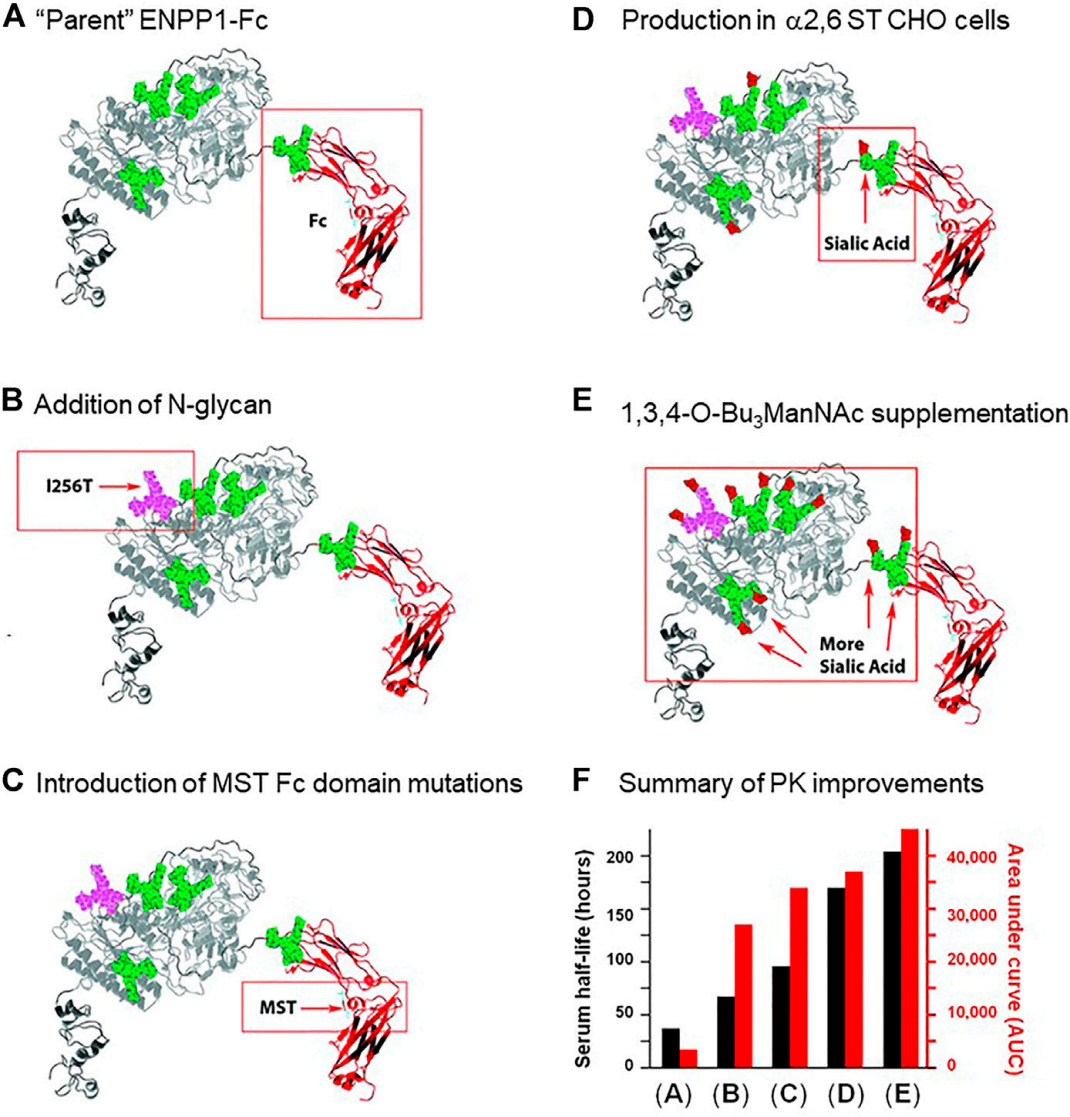
ENPP1 protein and glycoengineering. Improvements made to the pharmacokinetics of ENPP1 as reported by Stabach and coauthors (Stabach et al., 2021) are summarized in this figure. **(A)** First, in previous work (Albright et al., 2016), the enzyme was fused to the immunoglobulin Fc domain to increase protein recycling and serum recirculation through interactions with the neonatal Fc receptor (Albright et al., 2016); this “parent” construct had a serum half-life of 37 h and an AUC of 3,400 as depicted graphically in Panel **(F)**. **(B)** Addition of an N-glycan site was achieved through the I256T mutation to ENPP1 resulting in addition of the glycan to Asn254; this newly added N-glycan approximately doubled serum half-life and octupled the AUC value. **(C)** Mutation of Met, Ser, and Thr (MST) that increase affinity for the neonatal Fc receptor (Vaccaro et al., 2005) were introduced into ENPP1-Fc Fc’s domain, further improving both serum half-life and AUC. Finally, two approaches to increase sialylation including **(D)** production of ENPP1-Fc in α2,6-sialyltransferase overexpressing CHO cells and **(E)** supplementation of the culture medium with the sialic acid metabolic precursor 1,3,4-O-Bu_3_ManNAc sequentially further increased serum half-life (to a final value of 204 h) and the AUC value (to 45,000).

Despite the many benefits of subcutaneous administration, this method is limited in the volume that can be infused, and perhaps more importantly, the bioavailability of the therapeutic following injection. In one study, a glycoengineering strategy dramatically improved the bioavailability of subcutaneously delivered ENPP1-Fc. As a brief introduction, ENPP1 is ectonucleotide pyrophosphatase/phosphodiesterase 1, a blood enzyme whose deficiency results in generalized arterial calcification of infancy (GACI), a potentially lethal disease (Ferreira et al., 2021). Wild-type ENPP1 has a short serum half-life of ∼5 h when used for enzyme replacement therapy (ERT), necessitating thrice a day dosing in a mouse model of GACI for therapeutic effectiveness. Braddock’s research team first took a protein engineering approach by fusing an IgG Fc domain to ENPP1 (Martins et al., 2016). The resulting ENPP1-Fc construct had a substantially improved serum half-life of ∼37 h (Figure 3A) but nonetheless relatively modest bioavailability when delivered subcutaneously (Albright et al., 2016). By taking a glycoengineering approach and adding a fifth N-glycan site to ENPP1-Fc through an I256T mutation (Figure 3B; the methodology for adding N-glycans to therapeutic proteins is outlined in Section *5.1.3*), the serum half-life almost doubled (from 37 to 67 h; Figure 3F), while a surrogate measure of bioavailability, the cumulative “area under curve” (AUC) value for enzyme activity in the serum, increased dramatically by 794% from 3,400 to 27,000 units (Stabach et al., 2021).

As a caveat, the biochemical mechanism for the PK improvements for ENPP1-Fc remain incompletely defined; for example, unlike the “size matters” improvement when N-glycans were added to EPO (Figure 2), ENPP1-Fc is already a large-sized protein, making it unlikely that avoidance of kidney filtration was involved in its improved serum longevity. A straightforward explanation, such as increased enzyme activity for the I256T glycoform, was ruled out by measurements that show that the enzyme’s catalytic activity was affected negligibly (Stabach et al., 2021). Instead, it is plausible (but not experimentally verified) that reduced access of serum proteases to exposed protein surfaces protected by the newly-added glycan reduced degradation (Section 2.3.2), concomitantly increasing serum longevity. The increase in apparent bioavailability evidenced by the I256T glycoform’s dramatic AUC increase is also unexplained; a specific structure-activity response appears to be involved insofar as only one of over 50 glycovariants of ENPP1-Fc created in the study gained such a dramatically improved ability to effectively extravasate from the subcutaneous compartment into circulation (Stabach et al., 2021). At present, it is unknown if the mechanisms involved will apply to therapeutic proteins in general or whether they are unique to ENPP1-Fc.

#### 2.2.2 Hyaluronidase-Assisted Subcutaneous Delivery of Therapeutic Proteins

Unlike the addition of an N-glycan to ENPP1-Fc that serendipitously improved its PK properties, hyaluronidase provides a broader approach to facilitate the absorption and bioavailability of subcutaneously-delivered therapeutics. Hyaluronan contributes to inefficient bioavailability of subcutaneously-injected drugs by endowing the hypodermis with viscoelastic properties that prevent bulk fluid flow of liquids or the diffusion of drug molecules, in particular high molecular weight therapeutic proteins (Frost 2007). Recombinant human hyaluronidase (rHuPH20) enzymatically degrades hypodermal hyaluronan, helping to overcome this impediment for subcutaneous drug delivery (Frost 2007; Wasserman 2017; Liu et al., 2021). One example of hyaluronidase’s efficacy is for subcutaneous delivery of IgG to treat primary immunodeficiency diseases (PIDDs) where regular and prolonged bioavailability of antibodies is essential (Wasserman 2017). In a second example, hyaluronidase can degrade hyaluronan capsules associated with tumors, increasing the accessibility and effectiveness of anti-cancer drugs (Shuster et al., 2002; Whatcott et al., 2011; McAtee et al., 2014; Kohi et al., 2016; Maneval et al., 2020).

### 2.3 Metabolism: Enzymatic Modification and Intracellular Trafficking

The term “metabolism” (i.e., the “M” in ADME) is broadly defined in this article to include any host-mediated enzymatic modification of a therapeutic protein, including catabolic (Section 2.3.1) and biosynthetic (Section 2.3.2) activities, as well as aspects of intracellular trafficking (Section 2.3.3).

#### 2.3.1 Serum Sialylation and Desialylation

As mentioned above, a major determinant of serum longevity is the sialylation status of many types of therapeutic proteins through shielding from ASGP receptor-mediated clearance. Accordingly, efforts are made to fully sialylate therapeutic proteins as practical; for example, EPO produced in CHO cells has sialic acid occupancy of 70–85% up to as high as 99% [i.e., ∼22 sialic acids per molecule of darbepoetin alfa (Elliot et al., 2000; Egrie & Browne 2002)]. Once in circulation, sialidase present in the serum stochastically remove sialic acids over time. As proteins become less and less sialylated, the loss of this terminal sugar functions as a molecular clock leading to the clearance of older and damaged proteins by the ASGPR. More recently, the idea has emerged that biosynthetic sialylation can also occur in the serum; in particular, sialic acid is added to the Fc glycans of circulating IgG antibodies. In rodents, the sialylation of IgG N-glycans is linked to secreted ST6Gal1 produced by liver epithelial cells and CMP-sialic acid leached into the serum by degranulating platelets (Jones et al., 2016). This naturally-occurring precedent for post-production modification of immunomodulatory proteins (Johnson et al., 2013), along with the commercial availability of reasonably-priced sialyltransferases, has opened the door for cell-free glycoengineering of protein therapeutics (Section 5.1.2).

#### 2.3.2 Glycan Shielding of Protease Activity

Recently, the SARS-CoV-2 virus has provided a dramatic example of how glycans can shield a protease cleavage site. For this virus (Casalino et al., 2020; Gong et al., 2021), and others such as influenza (Tong et al., 2003), heavy glycosylation is advantageous for evading host immunity by shielding underlying immunogenic foreign epitopes of the viruses. Conversely, the furin protease cleavage site that mediates cell infectivity of SARS-CoV-2 is sterically shielded by nearby glycans, providing evolutionary pressure for reduced glycosylation (Zhang et al., 2021). Based on this precedent, the addition of glycans to therapeutic proteins has been considered for protection from proteases that cleave proteins during degradation, although a potential downside is loss of the protein’s biological function. Indeed, an original impetus for adding N-glycans to ENPP1-Fc (Figure 3) was to protect the enzyme from proteases (Stabach et al., 2021). Guan and others added an O-linked tri-mannose structure to insulin, enhancing its proteolytic stability and decreasing unwanted aggregation while maintaining biological activity (Guan et al., 2018). In addition to protection from protease degradation, N-glycans play multiple auxiliary roles in protein stability by protecting proteins from oxidation, aggregation, pH-induced damage, and thermal degradation (Qun & Qiu 2019).

#### 2.3.3 Intracellular Trafficking

Another way that glycosylation can affect protein degradation, although indirectly, is through intracellular trafficking. A naturally-occurring example is the impact of hybrid and complex N-glycans on the cell surface vs. lysosomal/endosomal targeting of endogenously-produced sodium potassium chloride cotransporter NKCC1 encoded by *SLC12A2* (Singh et al., 2015). A second example is that increased sialylation weakens the galectin lattice and directs the epidermal growth factor receptor (EGFR) for degradation instead of surface recycling (Lajoie et al., 2007; Mathew et al., 2016). The ability to modulate subcellular trafficking through N-glycan composition led to the use of glycoengineering to create successful enzyme replacement therapy for Gaucher disease (GD). For context, initial efforts in the 1970s to use unmodified human β-glucocerebrosidase to treat GD were unsuccessful because macrophages (the target cells in this disease) did not bind and internalize this enzyme when it was isolated from natural sources (Tekoah et al., 2013); it was later discovered that the enzyme’s inefficient uptake could be ameliorated through a glycoengineering approach.

Specifically, upon discovery that glycans with exposed terminal mannose residues facilitated macrophage uptake of β-glucocerebrosidase (Friedman et al., 1999; Sato & Beutler 1993), glycoengineered versions of this enzyme were created to treat GD. The first version made was imiglucerase (Cerezyme^®^) produced in CHO cells and modified enzymatically after production to expose mannose, resulting in ∼40–60% of exposed Man_3_ structures (Figure 1B). A second version, velaglucerase alfa (Vpriv^®^) is produced in human fibroblast carcinoma cells and achieves ∼100% exposed Man_5_-Man_9_ residues through treatment of the production cells with kifunensine, a mannosidase I inhibitor; this drug has ∼2-fold greater internalization into macrophages compared to imiglucerase, showing the importance of glycosylation in therapeutic efficacy (Brumshtein et al., 2010). Taliglucerase alfa (Elelyso^®^) is a third version of therapeutic β-glucocerebrosidase; it is produced in a carrot cell-based production system and achieves ∼100% exposed Man_3_ residues without *in vitro* processing or mannosidase inhibitors. Taliglucerase alfa has increased uptake into macrophages compared to imiglucerase (Shaaltiel et al., 2007), presumably because of its completely unshielded terminal Man_3_ groups. This example of multiple competing products to treat GD, using alternative methods to control glycosylation towards the common goal of exposed terminal mannose residues, illustrates the benefits of flexible biomanufacturing platforms that tailor glycosylation for individual diseases, as outlined in Section 5, below.

## 3 Impact of Glycosylation on Pharmacodynamics and Biological Activity

Here, in Section 3, we describe how biochemical interactions mediated through glycosylation affects a drug’s pharmacodynamic (PD) properties, which are defined as the body’s biological response to a drug [i.e., what the drug does to the body; the word comes from the Greek “pharmakon” meaning drug and “dynamikos” meaning power (Marino et al., 2021)]. Pharmacodynamic properties are broad, including receptor, cofactor, and ligand interactions as well as virtually all other biological activities of a protein (Marino et al., 2021). Therapeutic proteins fall into several categories; in this report, we cover therapeutic enzymes in Section 3.1, hormones in Section 3.2, and blood proteins in Section 3.3 (therapeutic antibodies are covered in Section 4), providing examples illustrating how glycans impact the PD properties of these drugs and how glycoengineering can improve therapeutic efficacy.

### 3.1 Enzymes

#### 3.1.1 Hyaluronidase

The biological activity of hyaluronidase, the enzyme that facilitates subcutaneous drug delivery through transient solubilization of hyaluronan in the hypodermis (Section 2.2.2), depends on glycosylation. Recombinant human hyaluronidase (rHuPH20) is heavily glycosylated with size N-glycan sites (Asn47, Asn131, Asn200, Asn219, Asn333, and Asn358) that are all modified with high mannose type N-glycans (Frost 2007; Liu et al., 2021) (Figure 1B). As discussed above (Section 2.3.3), high mannose structures target proteins for clearance *via* MRs; hyaluronidase’s glycosylation status also affects its biological activity, and by extension its PD properties (Liu et al., 2021). Specifically, PNGase removal of its N-glycans decreased enzymatic activity of rHuPH20 by ∼80% in an *in vitro* assay; in a corresponding *in vivo* test, aglycosylated rHuPH20 dramatically reduced trypan blue dispersion (a surrogate measure of drug diffusion) in a mouse model when compared with naturally-glycosylated enzyme (Liu et al., 2021). This study illustrated how N-glycosylation was necessary for rHuPH20 to solubilize host hyaluronan (i.e., a PD effect) for facilitating subcutaneous delivery of a second drug (i.e., a PK effect). As the complex interplay between such PK properties and PD endpoints becomes more widely appreciated, the growing toolkit to glycoengineer therapeutic proteins (Section 5) to optimize both endpoints is becoming increasingly important.

#### 3.1.2 Esterases

Esterases are a diverse family of enzymes that have several pharmaceutical roles. In some cases, reminiscent of the role of hyaluronidase in improving subcutaneous drug delivery, esterases augment the effectiveness of a second drug. For example, esterases activate pro-drugs such as the Alzheimer’s drug tacrine (Bencharit et al., 2003), doxazolidine carbamates (Burkhart et al., 2006), the breast cancer drug tamoxifen (Fleming et al., 2005), the influenza drug oseltamivir (Shi et al., 2006), and hexosamine analogs used in metabolic glycoengineering (Mathew et al., 2017; Sarkar et al., 1995; Wang et al., 2009) (Section 5.1.5). Esterases also detoxify narcotics such as cocaine and heroin (Pindel et al., 1997) as well as chemical warfare agents such as soman and tabun (Fleming et al., 2003). Finally, these enzymes are being investigated for the direct treatment of diseases such as Alzheimer’s (Greig et al., 2002; Nordberg et al., 2013; Saez-Valero et al., 2000), Similar to hyaluronidases, glycosylation modulates both the enzymes’ PK and PD properties (Kolarich et al., 2008; Schneider et al., 2013; Weikert et al., 1994; Xu et al., 2015). In particular, sialylation is important for prolonging serum circulation (Chitlaru et al., 1998; Fukami & Yokoi 2012) and glycosylation affects the catalytic activity of several esterases including human acetylcholinesterase (Velan et al., 1993), human carboxylesterase 1 (Arena de Souza et al., 2015; Kroetz et al., 1993), and human carboxylesterase 2 (Alves et al., 2016). In one example of how glycoengineering can improve esterases, a metabolic glycoengineering approach (Section 5.1.5) using 1,3,4-O-Bu_3_ManNAc to sialylation (Section 5.1.5) increased sialylation of glycans situated at the interface of trimeric units of carbosylesterase one; *in silico* modeling indicated that these glycans increased the stability of the multimeric, active form of this enzyme (Mathew et al., 2017).

### 3.2 Hormones: Hypoglycosylated Follitropins

In many cases, gain-of-glycosylation (e.g., increased sialylation or newly-added N-glycans) improve PK or PD properties of therapeutic proteins. In some cases, however, reduced glycosylation can be beneficial, as is illustrated by the follicle stimulating hormone (FSH). This hormone is produced in the anterior pituitary and travels through the circulation to gonodal cells where it interacts with FSH receptors (FSHRs) to promote follicle development in women and spermatogenesis in men (Daya 2004; Davis et al., 2014; Ulloa-Aguirre et al., 2018). Therapeutically, recombinant FSH or follitropins can substitute for naturally-occurring FSH deficiencies to treat infertility (Dias & Ulloa-Aguirre 2021).

Endogenous FSH consists of an alpha and beta subunit; both have two putative sites of N-glycosylation. The alpha subunit is consistently fully glycosylated with the beta subunit occupied with zero, one, or two N-glycans (Davis et al., 2014; Ulloa-Aguirre et al., 2018; Dias & Ulloa-Aguirre 2021). The alpha subunit of FSH plays a pivotal role in receptor interactions by engaging the receptor-ligand interface (Ulloa-Aguirre et al., 2018; Dias & Ulloa-Aguirre 2021). The importance of the glycosylation of the alpha subunit is illustrated by the deletion of one glycosite (at Asn78), which increases FSHR binding, while the removal of its other N-glycan (Asn52) decreases efficacy (Ulloa-Aguirre et al., 2018; Dias & Ulloa-Aguirre 2021). Similarly, removal of the glycosylation sites on the beta subunit of FSH yielded significantly greater bioactivity (Dias & Ulloa-Aguirre 2021). Overall, hypoglycosylated FSH 9- to 26- fold more active than its fully glycosylated variant but also experienced reduced *in vivo* half-life, presumably due to loss of α2,3-siaylation (Ulloa-Aguirre et al., 2018). These experiments completely removed N-glycans at each site and did not explore microheterogeneity leaving open the intriguing possibility that fucosylation, sialylation, increased glycan branching, or another property could be tuned to optimize the glycosylation profile for FSH to meet the dual but competing PK and PD requirements. Overall, FSH demonstrates how glycosylation can have complex effects on a therapeutic protein by augmenting one endpoint while undermining the other, reinforcing the need for versatile glycoengineering strategies to meet such competing demands.

### 3.3 Blood-Modulatory Proteins

Overall, therapeutic proteins are dominated by blood-acting or blood-modulatory proteins (e.g., EPO and ENPP1-Fc, discussed above and antibodies that largely function in the blood are the largest class of therapeutic proteins; Section 4). Another category of blood-regulatory proteins whose activity critically depends on glycosylation are clotting factors that need to be administered therapeutically for people with deficiencies in these proteins, such as hemophilia patients. Deglycosylation diminishes the conformational stability, activity, and macromolecular interactions of coagulation factor VIII [FVIII (Kosloski et al., 2009)] and decreases the effectiveness of factor XIII-B [FXIII-B (Hurjak et al., 2020)]. Based on the importance of glycosylation in blood clotting, efforts to produce coagulation factors in low-cost hosts (e.g., in plant cells, Section 5.3.4) to increase availability for patients are cognizant of the importance of maintaining appropriate glycosylation; this topic is discussed extensively in a review article by Top and coauthors (Top et al., 2019).

## 4 Therapeutic Antibodies

**FIGURE 4:**
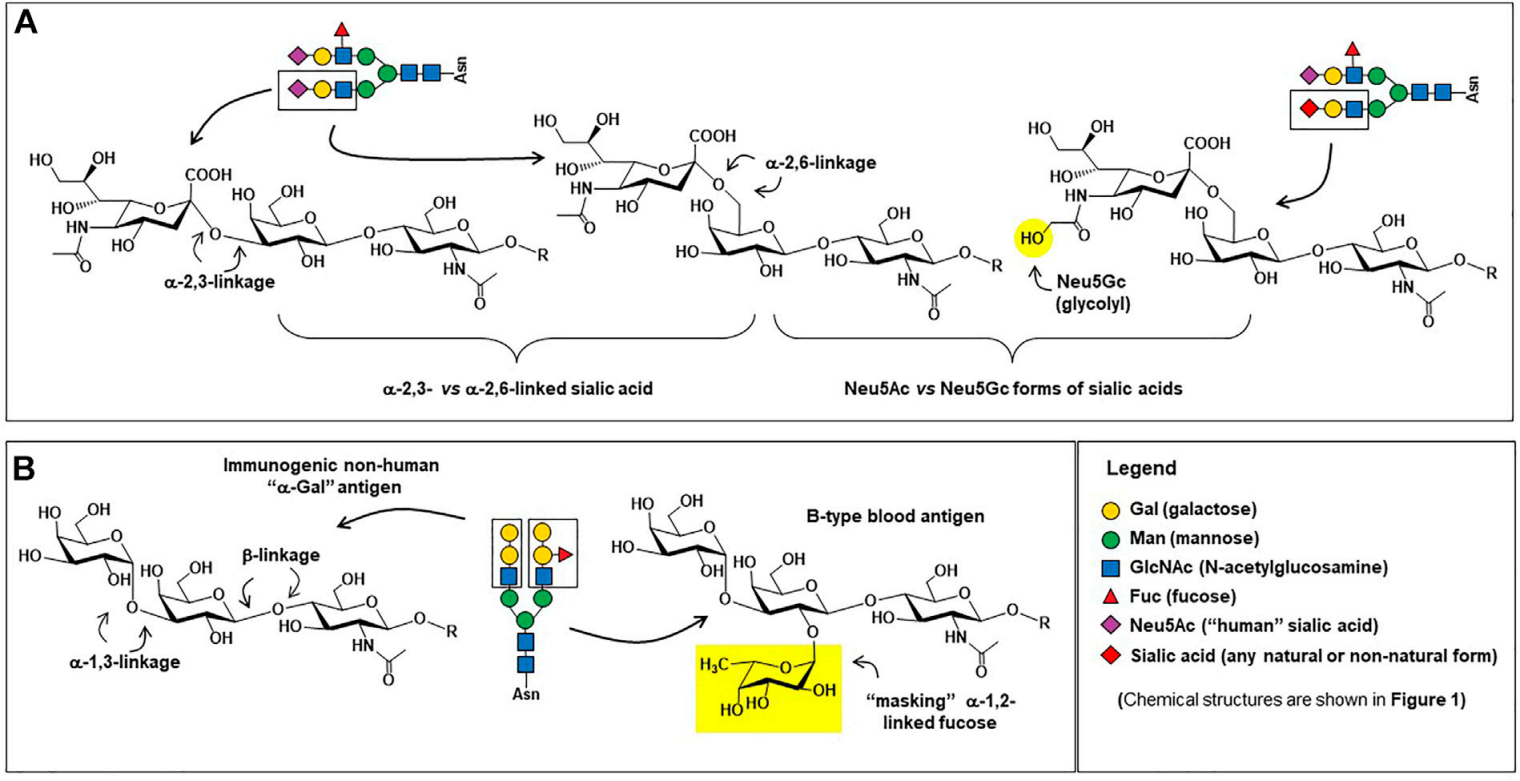
Carbohydrate epitopes relevant to therapeutic antibodies. **(A)** Sialic acid is found in human proteins in both α2,3-linkages (left) and α2,6-linkages (center); α2.6-linked sialic acid is critical for providing IgG antibodies with anti-inflammatory characteristics (Kaneko et al., 2006) whereas α2,3-linked sialic acid are effective at preventing ASPGR clearance (Ellies et al., 2002). The presence of the N-glycolylneuraminic acid (Neu5Gc, right) form of sialic acid on proteins produced in non-human mammalian cells can be pro-inflammatory (Tangvoranuntakul et al., 2003; Samraj et al., 2015), which may or may not be desired in a therapeutic protein. **(B)** The structure of the “α-Gal” trisaccharide epitope (left) is a major safety concern (Section 5.2.1); in human cells, the terminal alpha-linked galactose is not added to a glycan until the penultimate masking α1,2-linked fucose (right) is installed, preventing the synthesis of the “naked” immunogenic α-Gal epitope. Incidentally, the tetrasaccharide shown comprises the B-type blood antigen, whose present is a quality control parameter in IVIg therapy (Section 5.1.2).

Monoclonal antibodies are the largest class of biotherapeutics on the clinical market; in April 2021 the FDA approved its 100th monoclonal antibody product, GlaxoSmithKline’s PD1 blocker dostarlimab (Mullard 2021). The specificity, signaling versatility, and half-life of antibodies, all of which are modulated by glycosylation (Alter et al., 2018; Buettner et al., 2018; Irvine & Alter 2020), make them potent and highly sought therapeutics against a variety of diseases. Brian Cobb’s review article on antibody glycosylation (Cobb 2020) partitions the history of IgG glycosylation into two overlapping eras. The first era began in the 1970s when research uncovered how glycosylation contributed to the pro-inflammatory activities of IgG antibodies. Based on almost half a century of foundational knowledge, pro-inflammatory mAbs are now in clinical practice, mainly designed to destroy cancer cells; these efforts are described in more detail in Section 4.1. The second era of IgG glycosylation can be traced roughly to Jeffrey Ravetch’s group’s discovery that terminal α2,6-sialylation (Figure 4A) of IgG’s Fc glycans endowed these antibodies with anti-inflammatory properties (Kaneko et al., 2006). Efforts are underway to exploit these antibodies for intravenous immunoglobin (IVIg) and other therapies, as covered in Section 4.2. In the body, antibodies typically have either pro- or anti-inflammatory activities but their exquisite ability to bind to select targets—and by doing so inactivate the activity of the marker—has led to the creation of numerous blocking and neutralizing antibodies; as described in Section 4.3; to date this class of therapeutics has found great utility in cancer treatment by ablating the activity of oncoproteins and intense efforts are devoted developing broadly neutralizing antibodies for HIV-1. Finally, in Section 4.4 we outline glycoengineering approaches to increase the potency of antibody-drug conjugates.

### 4.1 Pro-Inflammatory Antibodies

As depicted in Figures 1D, 4A and described in detail elsewhere (Pereira et al., 2018; Wang et al., 2018; Zafir et al., 2013), glycan patterns on the conserved fragment crystallizable (Fc) region of IgG antibodies have significant effects on an antibody’s pro-inflammatory activities [e.g., antibody-dependent cellular cytotoxicity (ADCC), antibody-dependent cellular phagocytosis (ADCP), and complement-dependent cytotoxicity (CDC), discussed here in Section 4.1] as well as their anti-inflammatory activity (Section 4.2). Briefly stated, increasing elaboration of an Fc N-glycan with galactose, core fucose, and sialic acid increases anti-inflammatory activity, and antibodies designed to induce ADCC, CDC, and ADCP benefit from the absence of these monosaccharides (Buettner et al., 2018).

#### 4.1.1 Mechanism(s)

The majority of pro-inflammatory antibodies now in clinical use are designed to bind to tumor selective antigens and elicit downstream effector responses (Ząbczyńska et al., 2020) that kill the target cancer cells. Specific mechanisms of action include ADCC, ADCP, and CDC where ADCC is a type of immune reaction where the target cell becomes coated with the therapeutic antibodies and then is lysed by immune effector cells that include natural killer (NK) cells, macrophages, neutrophils, and eosinophils. ADCP utilizes a similar process but the effector cells, typically macrophages, phagocytose antibody-opsonized target cells instead of lysing them. CDC is mediated by IgG and IgM antibodies, which trigger the classical complement pathway to lyse the target cells upon binding of the C1q protein to the Fc region of Fcγ receptors. Naturally-occurring unbalanced glycosylation profiles can lead to and/or exacerbate pro-inflammatory ADCC and CDC in disorders, such as the destruction of thyroid tissue in Hashimoto’s thyroiditis (Ząbczyńska et al., 2020).

In general, with the effects of ADCC being the most thoroughly studied, sialic acid and core fucose inhibit these pro-inflammatory responses that often are desired in anti-cancer antibodies. Mechanistically, the glycan composition allosterically alters Fcγ receptor interactions, as reviewed in detail elsewhere (Zafir et al., 2013; Pereira et al., 2018; Wang et al., 2018). The IgG glycomes of human-derived antibodies are highly fucosylated, with afucosylated IgG ranging from only ∼1.3–19.3% in one study (Pucić et al., 2011); CHO cell-produced IgG has a similarly high fucose occupancy of 90% or more (Figure 1D). As discussed below (Section 4.1.2), the highly fucosylated glycoprofile of CHO cell produced antibodies has led to glycoengineering efforts to produce afucosylated mAbs to treat cancer *via* ADCC. By contrast, anti-cancer IgG antibodies produced in industry-standard CHO cells have an attractive pro-inflammatory profile insofar as 98% or more of the Fc domain N-glycans are asialylated; nevertheless, emerging evidence suggests that even residual levels of 2% or less sialic acid can have a profound anti-inflammatory effects (Section 4.1.3).

#### 4.1.2 Afucosylated Clinical Antibodies

Evidence that natural variations in Fc glycosylation impact IgG antibody activity spurred efforts to produce afucosylated therapeutic antibodies; for example, these antibodies have superior anti-HIV-1 activity (Ackerman et al., 2013). As of 2018, there were three FDA-approved afucosylated antibodies: Obinutuzumab (Gazyva^®^; targets CD20), Mogamulizumab (Poteligeo^®^; targets CC chemokine receptor 4, and benralizumab (Fasenra™; targets IL-5Rα), with more than 20 in clinical trials (Pereira et al., 2018). Since then, inebilizumab [Uplizna^®^; targets CD19 to treat neuromyelitis optica spectrum disorder (NMOSD) (Cree et al., 2019)] has been approved and ublituximab, which targets CD20 to treat multiple sclerosis and chronic lymphocytic leukemia is in the final stages of approval (Fox et al., 2021).

Of these afucosylated antibodies, obinutuzumab and mogamulizumab are both anti-cancer drugs where lack of fucosylation increases ADCC, ADCP, or CDC potency against tumor cells. For example, the afucosylated CD20-targeting drug obinutuzmab activates neutrophils and mediates phagocytosis more efficiently than rituximab, which is a normally fucosylated CD20-targeting mAb (Golay et al., 2013). By contrast, benralizumab blocks IL-5R signaling leading to ADCC-mediated depletion of IL-5Rα-expressing eosinophils (Kolbeck et al., 2010); in essence it is an anti-inflammatory mAb by leading to the death of excess immune cells to treat severe eosinophil asthma.

#### 4.1.3 Asialylated Clinical Antibodies

From a practical perspective, the glycan profile of IgG therapeutic antibodies produced in industry-standard CHO cells, superficially at least, has an attractive pro-inflammatory profile insofar as 98% or more of the drug copies are asialylated (Figure 1D). As a result, unlike multiple efforts to reduce fucosylation that already have been adopted for commercial biomanufacturing and received regulatory approval, efforts to reduce sialylation have lagged. Nevertheless, the importance of reducing even the residual levels of sialic acid in therapeutic antibodies was illustrated by a study of pertuzumab (Perjeta^®^, Genentech), a mAb that binds to HER2, blocking its dimerization and subsequent oncogenic signaling.

Although the mechanism of action of pertuzumab was originally described as a conventional blocking/neutralizing antibody (i.e., by blocking HER2 signaling in breast cancer), it also has pro-inflammatory activity *via* ADCC and CDC. To explore whether these activities could be augmented by desialylation, Luo and coworker enzymatically removed sialic acid from pertuzumab using neuraminidase, and observed an approximately five-fold increase in CDC and almost two-fold increase in ADCC (Luo et al., 2017). These increases were unexpectedly large, considering that the parent material was only ∼2.5% sialylated; the most plausible explanation for this result was that these residual levels of sialylation potently inhibit CDC and ADCC and neuraminidase treatment relieves this inhibition.

In theory, as pertuzumab illustrates, the complete removal of sialic acid (and fucose (Luo et al., 2017)) offers a way to improve the efficacy of anti-cancer mAbs by facilitating CDC and ADCC. However, as a counterargument to this strategy, pertuzumab has a substantial number of deleterious side effects, including diarrhea or constipation, hair loss, loss of neutrophils and red blood cells, hypersensitive allergic reactions, decreased appetite, insomnia, distorted taste perception, inflammation of the mouth and lips, rashes, and muscle pain. Therefore, in practice, increasing the pro-inflammatory potency of this drug could exacerbate these side effects, reducing patient tolerance and overall clinical efficacy. The “take-home” lesson is that in principle it could be beneficial to glycoengineer anti-cancer antibodies to increase their pro-inflammatory activities; in practice, however, these efforts must be balanced by the danger of exacerbating off-target side effects. The ability to precisely tune the pro-vs. anti-inflammatory properties of IgG antibodies has been demonstrated using chemoezymatic synthesis; for example, homogeneous glycoforms of cetuximab with Fab N-glycans with two, sialylated antennae and Fc N-glycans with no fucosylation or sialyation have been created. The end result was an antibody with equal binding affinity to EGFR and increased affinity to FcγRIIIa, generating stronger ADCC (Giddens et al., 2018).

### 4.2 Anti-Inflammatory Antibodies

#### 4.2.1 Mechanisms

As outlined above (Section 4.1 ), the role of fucose and sialic acid in the pro-inflammatory properties of therapeutic antibodies (Pereira et al., 2018)) are now well established. The flip-side to the necessary absence of both fucose and sialic acid for ADCC, ACDP, and CDC is that the presence of these sugars is beneficial–indeed, often required—for anti-inflammatory antibodies. For example, even residual levels of sialylation endow pertuzumab with potent anti-ADCC and anti-CDC properties (Section 4.1.3 above). To quickly summarize the role of these two sugars [along with galactose, which has a more modest effect (Buettner et al., 2018)], they function as a tunable on/off switch where their presence turns on the anti-inflammatory properties of antibodies.

#### 4.2.2 Immunoglobin G Therapy

Immunoglobulins from human donors are highly sialylated (from 20 to 60% site occupancy) compared to IgG antibodies produced in CHO cells (generally <2% and often <1%); accordingly, they have potent anti-inflammatory properties that can be attributed to their sialylation status (Li D. et al., 2021). As a result, polyclonal immunoglobulin provides a non-steroidal anti-inflammatory treatment safe for vulnerable patients, including children and pregnant women. More generally individuals with a broad range of autoimmune diseases including secondary hypogammaglobulinemia, recurrent infections, idiopathic thrombocytopenia purpura, Kawasaki disease, polyneuropathies, and graft versus host disease following organ transplantation (Barahona Alfonso & João 2016). Therapeutic immunoglobulin is typically administered intravenously as intravenous IgG (i.e., IVIg) therapy at up to 2 g/kg every few weeks to months (or, in rarer cases, subcutanoeus administration anti-inflammatory antibodies is achieved through co-delivery with hyaluronidase (Wasserman 2017)). With the continued growth of IgG therapy (Li D. et al., 2021), donor supply is projected to be insufficient, posing the quandary that CHO cell-produced recombinant IgG is poorly-sialylated (<2% overall and completely lacking in FcyR-modulating α2,6-sialic acids) and therefore lacking anti-inflammatory properties.

#### 4.2.3 Anti-Inflammatory Monoclonal Antibodies

Intravenous immunoglobulin (IVIg) therapy, by using pooled samples from multiple donors contains immunosuppressive antibodies against numerous epitopes and is broadly anti-inflammatory. An alternative approach is the development of anti-inflammatory monoclonal antibodies against single epitopes for the treatment of non-cancerous indications. These efforts began over 30 years ago with the development of the anti-TNFα infliximab to treat rheumatoid arthritis (Semerano & Boissier 2009). Within the next two decades, several anti-inflammatory monoclonal antibodies have been approved to treat not only rheumatoid arthritis but also Crohn’s disease, ulcerative colitis, spondyloarthropathies, juvenile arthritis, psoriasis, and psoriatic arthritis (Kotsovilis & Andreakos 2014). Indeed, four of the first five and the first seven of the first 10 FDA-approved mAbs were for anti-inflammatory indications (Lu et al., 2020). Although no longer as prolific as pro-inflammatory anti-cancer antibody drugs, anti-inflammatory monoclonal antibodies still comprise a substantial market share [e.g., including Orencia^®^, Humira^®^, Kineret^®^, Cimzia^®^, Enbrel^®^, Simponi^®^, and Remicade^®^ (Kotsovilis & Andreakos 2014)]. The success of these drugs is exemplified by Humira^®^, which, in 2018, had a market value of US$ 19.9 billion (Lu et al., 2020). Up to now, the lucky happenstance that industry-standard CHO cell production systems provide monoclonal antibodies with anti-inflammatory properties due to high fucosylation and residual 1–2% sialylation levels has allowed clinical anti-inflammatory antibodies to be successful. In the future, we predict that deliberate efforts to increase the anti-inflammatory nature of these drugs, e.g., through increased sialylation (Section 5.1), will make these drugs even more effective.

### 4.3 Blocking/Neutralizing Antibodies

#### 4.3.1 Mechanism(s)

In the body, the natural function of many antibodies is to have either pro- or anti-inflammatory activity (e.g., as discussed above in Section 4.1 and Section 4.2, respectively); many other antibodies, however, have blocking and neutralizing action (e.g., HIV-neutralizing antibodies). Naturally-occurring neutralizing antibodies typically function by binding to a virus or microbe, which can, at a minimum, negate the pathogen’s infectivity, and ideally target it for immune destruction. These antibodies provide precedent to exploit this class of molecules to, in theory, bind to any receptor and block its activity. These neutralizing antibodies, also commonly referred to as blocking antibodies, are currently the largest class of clinical FDA-approved protein therapeutics; indeed, multiple blocking antibodies exist for PD1/PDL1 (7 FDA approved drugs), CD20 (6), TNF (4), HER2 (4), CGRP/CGRPR (4), IL-7/IL-6R (4), IL23 p19 (3), EGFR (3) and CD19 (3) (Mullard 2021).

#### 4.3.2 Early Cancer Treatment Monoclonal Antibodies Were Blocking Antibodies

Immune checkpoint inhibitors are one of today’s most exciting cancer immunotherapies, evidenced by the largest category (7 of 100) FDA-approved monoclonal antibodies being in this category (Mullard 2021). Several of the first immunotherapies, particularly for anticancer mAbs, also were blocking antibodies, including rituximab (1997, CD20); trastuzumab (1998, HER2); alemtuzumab (2001, CD52); and cetuximab (2004, EGFR) (Lu et al., 2020). Of these, cetuximab is a notable example from almost 20 years ago that alerted the pharmaceutical industry and regulatory agencies to the importance of glycosylation when the α-Gal epitope posed a major safety concern, as discussed below (Section 5.2.1). Interestingly, despite its early development, cetuximab remains one of the few commercial IgG mAbs that have a non-canonical Fab region N-glycan (Ayoub et al., 2013; Janin-Bussat et al., 2013). In the future as the role of Fab glycans in auto-antibody responses and auto-immune diseases become better defined (van de Bovenkamp et al., 2016; Van de Bovenkamp et al., 2018), we predict that commercial mAb development will revisit this category of mAbs.

#### 4.3.3 HIV Neutralizing Antibodies

Human immunodeficiency virus 1 (HIV-1) remains an elusive and difficult-to-treat pathogen that causes acquired immunodeficiency syndrome (AIDS). The viral envelope’s negligible immunogenicity is attributed to its host-derived glycan shield similar to SARS-CoV-2 and influenza (Section 2.3.2). Antibodies against the virus primarily target the envelope spike glycoprotein (Env), the only viral protein on the virus’ surface, which is expressed in three form: gp120, gp140, and gp160 (Go et al., 2017; Heß et al., 2019; Seabright et al., 2019; Offersen et al., 2020; Wang et al., 2020). The Env protein is displayed sparsely on HIV-1, limiting the ability of antibodies to crosslink and elicit an immunogenic response to this virus. Nevertheless, certain individuals develop broadly neutralizing antibodies (bNAbs) against Env (Go et al., 2017; Seabright et al., 2019; Wang et al., 2020) that, although not providing a complete cure, do suppress most deleterious effects of HIV infection.

The capability of certain AIDS patients to produce bnAbs against HIV-1 spurred interest in mimicking these antibodies to produce effective vaccines. Engaging, or perhaps more precisely thwarting, glycosylation is critical for enhancing the immunogenicity of emerging HIV-1 vaccines. A longstanding difficulty in developing an effective bNAb vaccine is the notorious ability of HIV-1 to shift its glycosylation patterns (Wei et al., 2003), generating entirely new profiles in response to the adaptive immune response (Go et al., 2017; Offersen et al., 2020; Wang et al., 2020); a well-known example involves the N334 position on the Env protein (Seabright et al., 2019). As a counterpoint, bNAbs to Env function by recognizing glycosylation patterns that are conserved across clades of viral proteins, including atypical oligomannose structures (Seabright et al., 2019; Wang et al., 2020). Recent studies have focused on determining highly conserved glycoprofiles across viral strains, metabolic activities, and cell types (Wang et al., 2020) to facilitate bNAb-inducing HIV-1 vaccine development.

The previous two paragraphs laid out challenges facing natural immunity to HIV-1 infection, many of which result from viral glycosylation. To turn the tables on the virus, intriguing glycoengineering strategies have been directed towards treating AIDS. In one pioneering effort, Song and coworkers describe how the addition of an N-glycan to the HIV neutralizing antibody ibalizumab (Trogarzo^®^) improves its efficacy (Song et al., 2013). The added N-glycan helps fill “empty space” between the antibody and viral epitope, thereby increasing the binding interface and affinity. In this groundbreaking study, the glycan was limited a the Glc_2_Man_5_ structure (Figure 1B); in the future, follow-on glycoengineering efforts can further facilitate ibalizumab-Env binding interactions, resulting in even more potent neutralizing antibodies. (Strategies for attaining improved glycoforms towards these objectives are provided in Section 5 of this report.)

### 4.4 Antibody-Drug Conjugates

Antibodies are attractive drug delivery vehicles because their binding specificity allows them to deliver payloads with minimal off-target toxicity. As such, a variety of methods have evolved to directly link a drug of interest to an antibody, thus forming antibody-drug conjugates (ADCs). Conventional chemical conjugation of drug payloads typically utilize the amines of lysine or thiols of cysteine residues present in the amino acid sequence of the antibody (Qasba 2015; Tang et al., 2019). This approach results in heterogeneous ADCs with greater susceptibility to aggregation, decreased antibody stability, or cytotoxicity that together pose barriers to effective clinical use and increase regulatory scrutiny.

**FIGURE 5:**
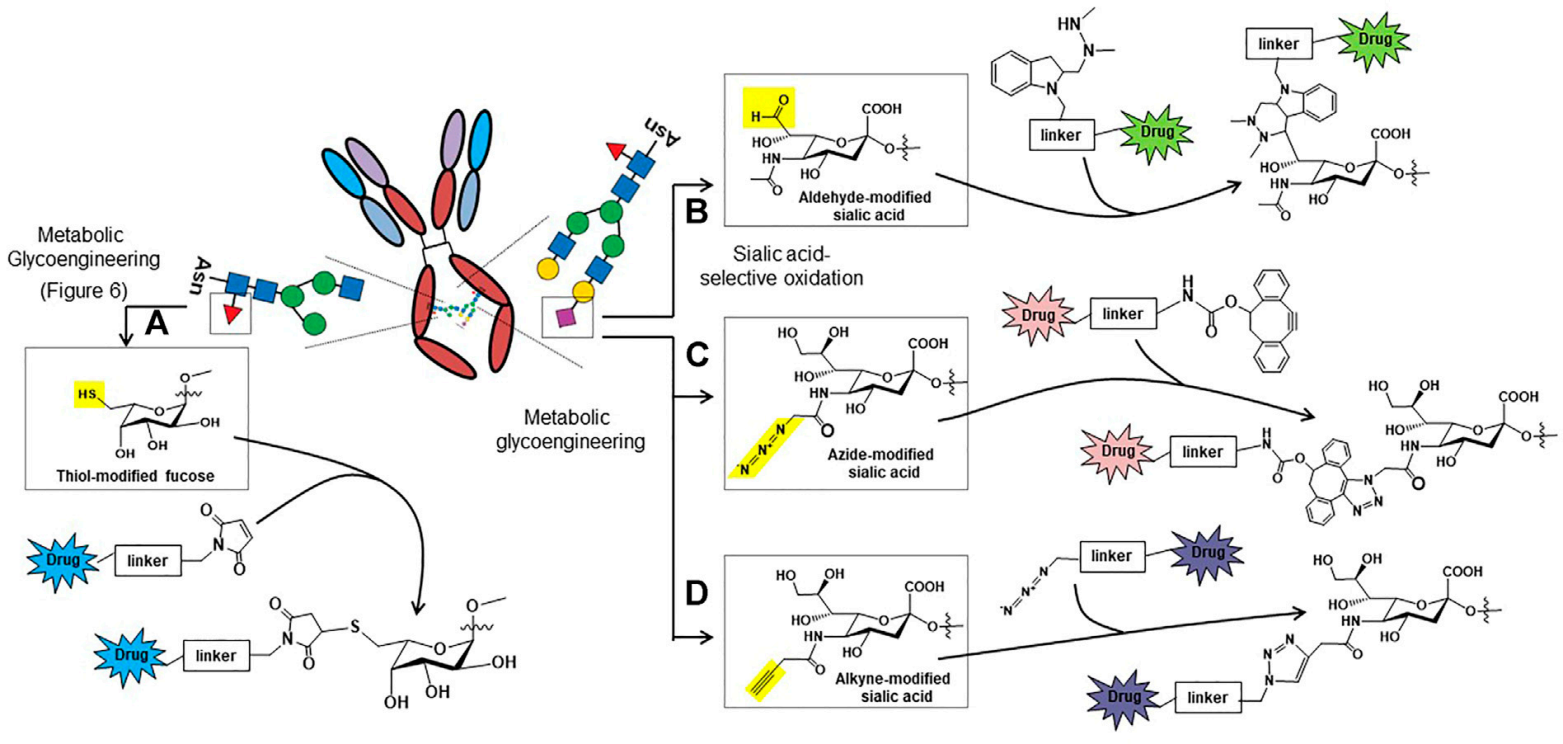
Glycosylation-based antibody-drug conjugate (ADC) ligation strategies based on chemically-modified fucose (A) or sialic acid (B, C, and D). **(A)** Thiols can be installed into non-natural fucose using metabolic glycoengineering and used as “chemical handles” to ligate drug molecules to the Fc domain glycans of antibodies using thiol-reactive maleimides (Okeley et al., 2013). **(B)** Aldehydes can be selectively introduced into sialic acids by oxidizing the C8-OH groups; the aldehyde then can be conjugated to drugs using the hydrazino-*iso*-Pictet-Spengler (HIPS) reaction (Drake et al., 2014). **(C)** Metabolic glycoengineering can be used to install azido-sialic acids into glycans (Saxon & Bertozzi 2000), which can then be used to conjugate drugs to the antibody using dibenzocyclooctyne (DIBO) conjugation reactions (Li et al., 2014). **(D)** Alkyne groups can also be introduced into sialic acids through metabolic glycoengineering, which can then be conjugated using conventional copper catalyzed click chemistry (Du et al., 2009; Hong et al., 2010).

**FIGURE 6:**
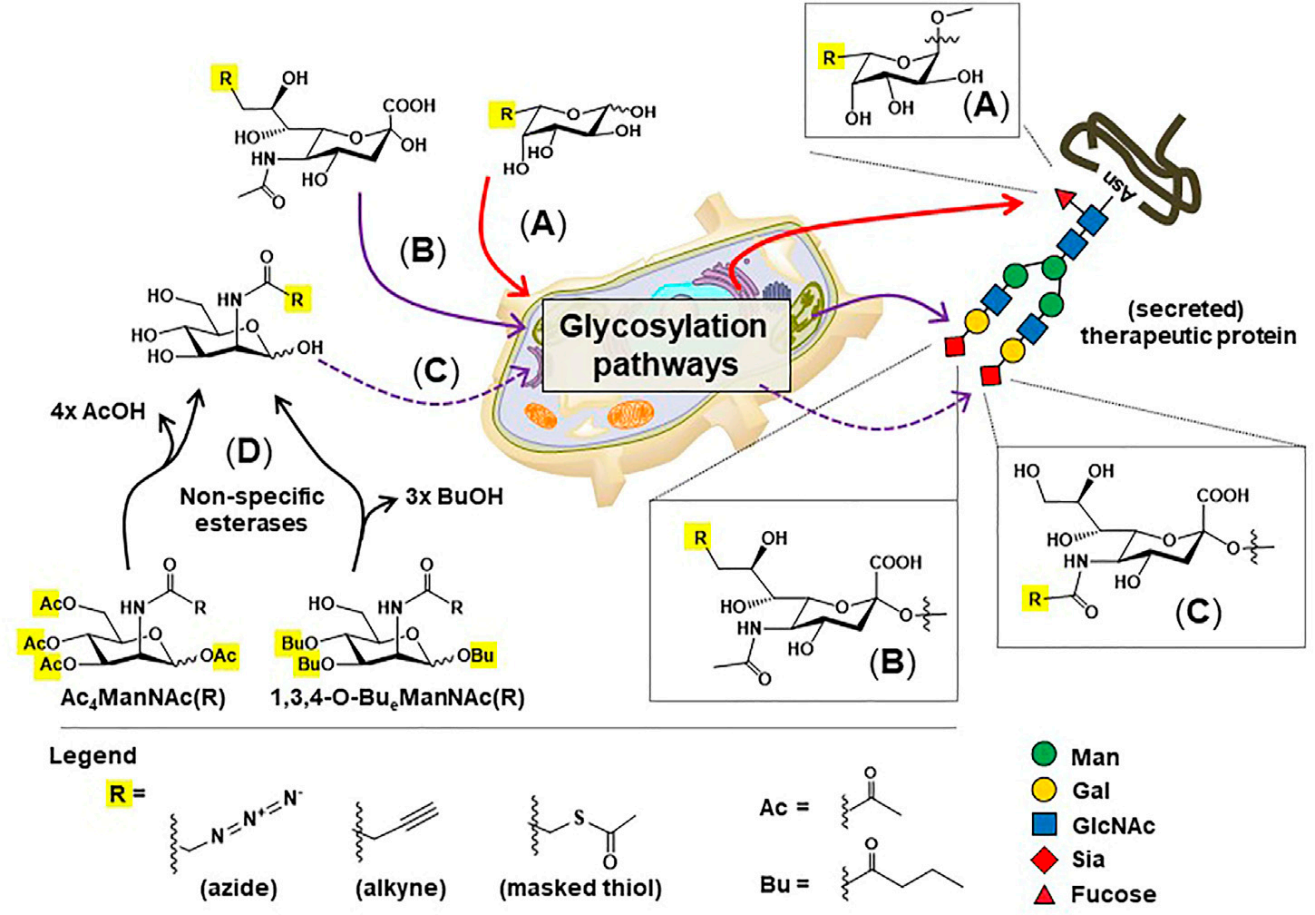
Overview of metabolic glycoengineering (MGE). Non-natural monosaccharide analogs capable of installing “chemical handles” into the N-glycans of therapeutic proteins include: **(A)** C6-modified fucose **(B)** C9-modified sialic acids, and **(C)** C2-modified ManNAc analogs, which are converted to N-acyl (C5) modified sialic acids before installation into N-glycans. **(D)** “High-flux” esterase-protected ManNAc analogs are now widely employed in MGE experiments to increase cell uptake and reduce the concentrations required for media supplementation from 30 to 75 mM (Yarema et al., 1998) to 100 μM or less (Jones et al., 2004; Kim et al., 2004; Almaraz et al., 2012).

These pitfalls have spurred researchers to create active, homogenous ADC populations with one such class of these drugs known as glycosite-specific ADCs (gsADCs) (Tang et al., 2019). These glycoengineering strategies take advantage of the conserved, biantennary N-glycosylation site present at the asparagine 297 residue of the CH2 regions of the Fc domain. One strategy uses metabolic glycoengineering to install thiol-modified fucose in Fc domain glycans (Figure 6A), which can be used as a chemical handle for drug conjugation (Figure 5A, (Okeley et al., 2013)). Another chemical method for site-specific chemical conjugation to Fc glycans involves mild periodate oxidation (Jourdian et al., 1971; Peters & Aronson Jr 1976), which selectively introduces aldehyde groups into sialic acids (Figure 5B); a downside of this approach is the low sialylation of Fc glycans, often 2% or lower. A strategy using non-natural ManNAc analogs to increase flux through the sialic acid pathway (Figure 6) and simultaneously install bioorthogonal chemical functional groups such as azides (Figure 5C) and alkynes (Figure 5D) provides additional options to create gdADCs.

## 5 Considerations for the Design and Production of Glycoengineered Therapeutic Proteins

So far, this report outlined various ways that glycosylation controls the pharmacokinetics, pharmacodynamics, and overall clinical efficacy of therapeutic proteins. Knowing this, biomedical researchers and the pharmaceutical industry are increasingly aware of the importance of controlling the glycosylation of therapeutic proteins using glycoengineering strategies summarized in Section 5.1. Ultimately, the production of glycoengineered proteins depends on glycocompatible production systems, which today are focused on CHO cell biomanufacturing (Section 5.2. Finally, arguments that the current industry-standard CHO cell platform is stifling innovation, especially with respect to glycosylation, are leading to the development of alternative cell-based production platforms (Section 5.3)

### 5.1 Glycoengineering–Methods and Approaches

#### 5.1.1 Glycoengineering of Proteins Isolated From Natural Sources to Increase Their Effectiveness

The clinical use of therapeutic proteins pre-dated today’s recombinant protein production technologies with early generations of these drugs obtained from natural sources; insulin is a well-known example initially derived from bovine and porcine pancreases. Additional examples from the current report include hyaluronidase obtained from mammalian sperm, β-glucocerebrosidase isolated from human placenta (Deegan & Cox 2012), blood coagulation and clotting factors obtained from human plasma, and FSH prepared from human urine from postmenopausal women. In some cases, exemplified by β-glucocerebrosidase, glycoengineering was a critical enabling technology to turn this enzyme into a useful drug by installing high mannose N-glycans (Figure 1B) that enabled macrophage uptake to treat GD. In other cases, illustrated by FSH, the complex role of glycosylation is still being unraveled. For example, certain glycoforms can be beneficial for PD properties while detrimental for PK properties and vice versa; once a fuller understanding is in hand, glycoengineering strategies can be applied to improve this type of therapeutic protein. There is even evidence that the few non-glycosylated therapeutic proteins can benefit from glycoengineering, for example, insulin with three newly-added N-glycans has improved resistance to proteases, potentially opening the door to oral dosing (Guan et al., 2018).

Chemoenzymatic synthesis, which combines chemical synthesis with the use of enzymes such as glycosyltransferases, glycosidases, lipases, and glycosynthases, is a powerful method for the synthesis of complex glycans and glycoproteins (Muthana et al., 2009; Wang et al., 2019; Ma et al., 2020b; Zeng et al., 2022). In addition to building new glycans, chemoenzymatic methods can be used to remodel glycans on antibodies and other glycoproteins, thus improving glycoform homogeneity (Wang et al., 2019). Additionally, this method removes the need for protection and deprotection of peptides that occurs in purely chemical synthesis (Zeng et al., 2022). Because this approach combines both the selectivity of enzymatic reactions and the flexibility of chemical glycan synthesis, it provides a facile method for the synthesis of complex polysaccharides, heparin sulfates, glycoproteins and glycolipids that are difficult to synthesize homogeneously *via* other methods (Muthana et al., 2009). For example, chemoenzymatic glycan remodeling of IgG antibodies can be employed to produce glycosite-specific antibody-drug conjugates (Zeng et al., 2022).

#### 5.1.2 Cell-Free Methods to Modulate Glycosylation: IVIg Therapy as a “Case Study”

Immunoglobulin used in IVIg therapy illustrates how isolation of therapeutic proteins from natural sources (e.g., human blood donors) is a cumbersome and inefficient process. Depending on the manufacturer, 1,000 to 100,000 donor samples are pooled to purify and concentrate IgG to 50–100 mg/ml with preparations typically still containing residual levels of IgE, IgM, and IgA antibodies at ≤ 700 μg/ml. The pooled samples are screened for viral contamination (Hep B, Hep C, and HIV) and monitored for conformance to an acceptable titer of ABO blood type-recognizing antibodies to reduce risk of hemolytic reactions in the recipients (Barahona Alfonso & João 2016). At the end of this cumbersome process, sialylated IgG antibodies can be as low as 15% of the total, resulting in less-than-optimal anti-inflammatory potential.

The enticing prospect of increasing the potency of immunoglobulin therapy by enhancing the α2,6-sialylation (Figure 4A) of donor IgG has been apparent for ∼15 years (Kaneko et al., 2006). Li and coauthors summarize several attempts to increase sialylation (Li D. et al., 2021), one of the first efforts involved the use of *Sambucus nigra* agglutinin (SNA) affinity chromatography to prepare IVIg to treat rheumatoid arthritis (Sudo et al., 2014). Taking a different approach, chemoenzymatic strategies to improve IgG sialylation incubate pooled IgG samples with α2,6-sialyltransferase in the presence of CMP-sialic acid (the enzyme’s co-substrate). In some cases, the IgG is pretreated with neuraminidase to remove non-inflammatory α2,3-sialic acids and, in other cases, the sialylation reaction is done in the presence of β1,4-galactosyltransferase and UDP-galactose to install the penultimate galactose required for terminal sialylation (Anthony et al., 2008; Washburn et al., 2015; Bartsch et al., 2018). Another enticing approach, pioneered by Lai-Xi Wang’s group, is to use transglycosidases to remove existing Fc domain N-glycans and enzymatically replace them with homogenously sialylated glycans (Li et al., 2017; Giddens et al., 2018; Wang et al., 2019).

These post-production glycoengineering strategies have successfully improved the efficacy and potency of immunoglobulin therapy; for example, a 0.1 g/kg dose of SNA-enriched IVIg is as effective as 1.0 g/kg of unfractionated drug (Kaneko et al., 2006). A major pitfall, however, is that these methods can only be performed on the milligram to Gram scales (or optimistically, on a kilogram scale) based on the expense of the lectins, glycosyltransferases, and nucleotide sugar donors involved (Li D. et al., 2021). Considering that worldwide consumption of IVIg is over 100 tons per year, post-production chemoenzymatic glycoengineering strategies remain niche technologies not yet applicable to large scale preparation of this drug. This case study illustrates the need for versatile cell-based production systems for manufacturing of glycoengineered protein therapeutics where cells produce expensive reagents such as glycosyltransferases and nucleotide sugar donors essentially “for free” (i.e., they are produced by cellular metabolism).

#### 5.1.3 Cell-Based Production of Recombinant Glycoproteins

With a few notable exceptions (e.g., IgG antibodies for IVIg therapy that are isolated and purified from natural sources), today’s therapeutic proteins are produced in cell-based systems. Production in living cells became an option with the maturation of DNA cloning technologies in the late 1970s and early 1980s that enabled recombinant techniques for protein expression. Benefits for cell-based production of recombinant proteins are numerous including theoretically limitless supplies of the therapeutic, the ability to humanize products by altering the amino acid sequence to avoid immunogenicity and increase productivity, easier purification, and the avoidance of potential pathogens and immunogens from non-human sources. Equally important and most germane to this article, cell-based production systems can be customized to provide beneficial glycosylation patterns as discussed in detail in Section 5.2 for CHO cells and in Section 5.3 for emerging alternative production systems.

#### 5.1.4 “Building in” N-Glycan Sites

Natural N-glycosylation machinery recognizes a consensus sequon (Asn–X–Ser/Thr, where X is any amino acid except proline), and initiates glycosylation with the addition of the LLO 14-mer (Figure 1A, Step 1) to the nitrogen atom of the asparagine side chain. In theory, the installation of new N-linked glycans into a protein of interest can be achieved by introducing amino acid substitutions that yield this sequon. In practice, this sequon is a necessary, but not sufficient, condition for successful N-glycosylation because, for example, the built-in glycan must not interfere with protein folding. Even if a target protein is successfully glycosylated, the required amino acid substitution(s) or neoglycan may lead to structural alterations that deleteriously affect PK, PD, or therapeutic efficacy. In the past, efforts to add N-glycan sites to therapeutic proteins used a trial-and-error process. For example, two decades ago when darbepoetin alfa was designed, several dozen variants of recombinant human EPO containing one or more new sites for N-glycan attachment were evaluated (Elliot et al., 2000; Egrie & Browne 2002). More recent approaches for glycosylation site installation combine structural information with rational and computational design approaches to more efficiently design functional and efficacious constructs.

To design new glycosylation sites, a script with a sliding window evaluation of every amino acid triplet can be employed to identify all possible sites for insertion of an N-glycan by modifying existing amino acid sequences to the Asn–X–Ser/Thr consensus sequence. This method quickly identifies single and double amino acid substitutions that yield potential sites for N-linked glycosylation. Ideally, the sequence change should be minimal (i.e., a single amino acid mutation is ideal), to offer the highest probability that the protein remains functional. Once potential sites for N-glycans have been identified, further *in silico* evaluation can help guide specific glycovariants to be made experimentally. Online tools such as the NetNGlyc Server, an N-linked glycosylation prediction site (Gupta & Brunak 2002), can be used to estimate the likelihood that each of the possible engineered glycosylation sites will be successfully glycosylated. Sites with low likelihood of glycosylation (<0.55) can be disregarded before proceeding; in our experience, most sites with prediction frequencies of >0.70 or more are successfully glycosylated (Saeui et al., 2020).

Using structure design tools, such as the PyMOL mutagenesis wizard or the Rosetta software package, each neoglycosylation site within a glycoengineered protein can be modeled to ensure that desired features of the protein structure are maintained. First, the glycosylation site should be solvent-exposed and not be buried within the interior of the protein. Second, the glycosylation site should be positioned to avoid steric interference of attached glycans with important domains of the protein. For example, if the therapeutic protein is an enzyme, the glycan should not interfere with substrate access to its active site; this was a design feature for ENPP1-Fc (Figure 3), where built-in glycans did not comprise substrate binding or catalysis (Stabach et al., 2021). If the protein is a cytokine, hormone, growth factor, or antibody, the glycan should not interfere with the therapeutic protein’s binding to partner proteins. In certain cases, glycan-based steric factors can be advantageous to the protein’s function. For example, the increased size resulting from installed glycans in darbepoetin alfa leads to decreased kidney filtration and extended pharmacokinetic half-life (Section 2.1.1). As another design feature, also considered for glycoengineered ENPP1-Fc, novel N-glycans can be situated to block protease access to vulnerable surfaces of the enzyme (Stabach et al., 2021). Finally, the addition of new glycans can improve binding affinity through their introduced ionic, van der Waals, or entropic forces as exemplified by improved affinity of an HIV-neutralizing IgG antibody to gp120 upon addition of a non-canonical glycan (Song et al., 2013). Regardless of the glycoengineering objective, candidate proteins must be individually evaluated to ensure that their functional activity is as desired.

The use of *in silico* tools in combination with structural information can be used to rationally design N-linked glycosylation cites with the goal of maintaining, or even enhancing, the activity of the target protein. In cases where a solved structure is unavailable, *in silico* structure prediction tools can be leveraged to generate theoretical protein structures and guide design of theoretical N-linked glycosylation sites. Various computational tools have emerged to generate protein structures using homology-based and/or *de novo* modeling in place of directly resolving the protein structure (Kuhlman & Bradley 2019; Jumper et al., 2021; Kryshtafovych et al., 2021). Furthermore, recent advances in modeling glycans themselves can be incorporated into the design process, to provide additional information about how the structure and activity of a protein may be impacted by the glycans themselves (Labonte et al., 2016; Li M. et al., 2021). In summary, the ability to predict both protein and glycan structure using computational tools empowers many glycoengineering approaches where structural information is lacking.

#### 5.1.5 Metabolic Glycoengineering: Further Control of Glycan Chemistry

Metabolic glycoengineering (MGE, Figure 6) is a method pioneered approximately 40 years ago when Brossmer and others developed chemically-modified sialic acid analogs, including bulky moieties such as fluorophores, that could be enzymatically installed into glycans (Gross & Brossmer 1988; Gross et al., 1989; Gross & Brossmer 1995). Subsequent advances in the 1990s and 2000s include the Reutter group’s demonstration of MGE in living cells and animals (Kayser et al., 1992b; Keppler et al., 2001; Wratil et al., 2016); the Bertozzi group’s development of analogs with chemical functionalities not normally found on cells, thereby allowing bioorthogonal chemoselective ligation reactions (Mahal et al., 1997; Saxon & Bertozzi 2000; Prescher et al., 2003); the extension of MGE to biosynthetic pathways beyond sialic acid including fucose (Sawa et al., 2006; Okeley et al., 2013), GlcNAc (Vocadlo et al., 2003; Du et al., 2009), and GalNAc (Kayser et al., 1992a; Boyce et al., 2011); as well as efforts to incorporate high-reactivity chemoselective reaction partners including ring-strained cyclooctynes (Baskin et al., 2007; Ning et al., 2010) and tri- or tetrazines (Kamber et al., 2019; Agatemor et al., 2020).

Today, MGE technologies have matured to the point where they comprise an attractive toolkit for cancer treatment (Agatemor et al., 2019; Wang & Mooney 2020), and increasingly, for other conditions such as enhancement of neuronal differentiation for spinal cord and brain regeneration (Sampathkumar et al., 2006; Du et al., 2021; Du et al., 2022) Specific to therapeutic proteins, MGE can be used in various ways. For example, MGE can be used to endow antibodies with “chemical handles” into antibodies by replacing core fucose with thiol-modified residues and terminal sialic acids with their azido-modified counterparts (Section 4.4; Figure 5). In theory, introduction of non-natural sialic acids into IgG Fc domain glycans can achieve an antibody-to-drug ratio of four if both glycans are fully sialylated, biantennary structures; in practice, however, the low site occupancy of sialic acid on Fc domain glycans hinders the use production of high valency ADCs.

A variation of MGE can help overcome suboptimal levels of sialic acid by improving the sialylation of IgG Fc domain glycans and therapeutic proteins in general. Briefly, “high-flux” MGE analogs began with peracetylation where the ester-linked acetyl groups rendered the sugars more lipophilic, facilitating diffusion into cells (Lemieux et al., 1999; Sarkar et al., 1995; Yarema et al., 2001). Upon entry into a cell, non-specific esterases remove the acetate groups, allowing the “core” monosaccharide to enter its targeted biosynthetic pathway (Mathew et al., 2012; Wang et al., 2009). Our team discovered that tri-butanoylated hexosamines, exemplified by 1,3,4-O-Bu_3_ManNAc (Figure 6D), provide even higher flux into biosynthetic pathways, increasing sialylation with high efficiency (Aich et al., 2008; Almaraz et al., 2012). This analog increases the sialylation of therapeutic proteins including IgG antibodies (Yin et al., 2017), EPO (Mertz et al., 2020) and ENPP-1 (Stabach et al., 2021). In the case of ENPP1-Fc, production with 1,3,4-O-Bu_3_ManNAc increased serum half-life from 170 to 204 h and the AUC from 37,000 to 45,000 (Figures 3E,F).

### 5.2 Current Therapeutic Protein Biomanufacturing Overwhelmingly Uses CHO Cells

Chinese hamster ovary (CHO) cells have become the workhorse biomanufacturing platform for therapeutic proteins over the past 2 decades. Because of the importance of these cells, we describe their safety qualifications (Section 5.2.1), limitations (Section 5.2.2), and efforts towards overcoming these pitfalls by using genetically modified CHO cell variants with altered glycosylation capacities (Section 5.2.3).

#### 5.2.1 Safety Issues–Exemplified by the α-Gal Trisaccharide Immunogenic Epitope

Chinese hamster ovary cells have become the “go-to” cell line for biomanufacturing therapeutic proteins for several reasons, including efficiency, cost-effectiveness, and—historically—for safety reasons. Historically, CHO cells have been used for recombinant protein production since the 1980s based on several advantages, including their ability to produce relatively large amounts of glycoproteins, their lack of human pathogens, and their ability to approximately replicate human glycosylation patterns (Ma et al., 2020a). Over the past decade or so, production has coalesced around CHO cells for safety/regulatory reasons after pioneering anticancer antibodies severely harmed patients in early clinical testing. In particular, in 2004 cetuximab (Erbitux^®^)—a blocking antibody that inhibits the epidermal growth factor receptor (EGFR) and is used to treat metastatic colorectal cancer and head and neck cancer -- triggered anaphylaxis in cancer patients, resulting in several deaths (Friedman 2008). The affected patients had pre-existing IgE antibodies against galactose-α-1,3-galactose (i.e., “α-Gal” Figure 4B) generated by lone star tick bites; subsequent anaphylaxis was elicited by the presence of α-Gal on Erbitux^®^ produced in murine SP2/0 cells (Steinke et al., 2015). This incident raised awareness that CHO cells, which do not make α-Gal are safe host cells for biomanufacturing of therapeutic proteins helping these cells gain widespread regulatory acceptance.

#### 5.2.2 Limitations/Drawbacks of CHO Cells

Chinese hamster ovary cells have glycosylation patterns that are generally regarded as safe (i.e., they lack the hyper-immunogenic α-Gal epitope) but they do have drawbacks. For example, they lack α2,6-sialyltransferase activity, making them inappropriate production hosts for potently anti-inflammatory antibodies. Another pitfall is that CHO cells produce the Neu5Gc form of sialic acid (Figure 4B) (Hokke et al., 1990); although only weakly immunogenic, its presence in therapeutic proteins has raised caution (Ghaderi et al., 2010; Ghaderi et al., 2012). Despite these shortcomings, CHO cells currently produce ∼90% of therapeutic antibodies including virtually all newly-approved drugs. One reason why CHO cells are dominant is because of their acceptance by regulatory agencies, which can be regarded as a positive feature but also has its drawbacks. For example, quoting from Burnett and Burnett (Burnett & Burnett 2020):

“As promising as technology may be, drug companies are unwilling to risk the huge sums of money required to get a new product approved by the large drug approval administrations (e.g., the FDA or EMA) if there is already a proven alternative expression system with regulatory approval. This economic constraint has a stagnating effect on the pharmaceutical industry, limiting the scale of progress and development of new drug production technologies.”

Despite the stifling influence of regulatory agencies that have helped embed CHO cells as the go-to cell line for biomanufacturing, efforts continue to develop alternative production platforms. These efforts are not primarily driven by glycoengineering concerns but they often represent substantial departures from standard glycosylation patterns inherently produced by CHO cells. As such they face regulatory hurdles but also provide opportunities to tune glycosylation to improve the efficacy of therapeutic proteins.

#### 5.2.3 Genetically Modified CHO Cells

Before describing major departures from CHO cells (e.g., the use of bacteria, plant cells, yeast, and insect cells for biomanufacturing, Section 5.3), we cover “baby steps” being taken to rectify glycosylation deficits in CHO cells, or more positively, to endow them with enhanced glycosylation capabilities. Mammalian cells have 250 or more glycogenes, the majority are glycosyltransferases present in the Golgi, the last stage of glycan production (Tariq et al., 2018). The potential for genetic control of glycosylation in CHO cells was demonstrated almost 30 years ago by a library of lectin-selected, mutant sublines developed by Pamela Stanley’s research group (Stanley et al., 1996; Stanley & Patnaik 2005).

Today, advances in nucleic acid gene-editing techniques including zinc finger nucleases (ZFNs), transcription activator-like effector nucleases (TALENs), and clustered regularly interspaced short palindromic repeats with Cas9 protein (CRISPR/Cas9) facilitate precise, stable, and systematic engineering of the glycosylation capabilities of mammalian cells (Narimatsu et al., 2021; Wang et al., 2019). One example is the over-expression of α2,6-sialyltransferase (ST6) in non-human cell lines such as CHO cells (Yin et al., 2015), which have been used to produce EPO and IgG antibodies (Mertz et al., 2020; Yin et al., 2015) as well as ENPP1-Fc with improved sialylation and PK properties [Figure 3D, (Stabach et al., 2021)]. ST6 over-expression increases overall sialylation and results in a humanized α2,6-sialylation profile in CHO cells. In addition to over-expression of glycogenes to improve CHO cells as production hosts for therapeutic proteins, it can be advantageous to knock out other glycogenes. Indeed, the first glycogene KO’d for biomanufacturing involved a tour-de-force effort in CHO cells where two rounds of targeted homologous recombination ablated the two allelic copies of the α6-fucosyltransferase (Fut8) gene (Narimatsu et al., 2021). These efforts have reached fruition with several afucosylated therapeutic antibodies now in clinical use (Section 4.1.2).

### 5.3 Additional Cell-Based Options for Biomanufacturing Therapeutic Proteins

The limitations of CHO cells for biomanufacturing (Section 5.2.1) have kept alive efforts to develop additional cell lines as production platforms. Here in Section 5.3 we describe cell systems used to produce at least one, and often several, FDA-approved therapeutic proteins; each is discussed briefly providing a perspective on the system’s current use and future prospects with an emphasis each system’s glycoengineering capabilities.

#### 5.3.1 Human Cells

By definition, production of therapeutic proteins in human cells provides the drugs with humanized glycosylation, including features such as α2,6-sialylation lacking in CHO cells and, unlike mouse cells, a lack of α-Gal that enhances safety. Downsides of production include the high cost and potential safety concerns of animal products used in production (e.g., fetal bovine serum is generally required for the culture of human cells, opening the door to xenopathogen contamination) to low productivity (typically one to ∼100 s mg/L) (Dumont et al., 2016). Nonetheless there are five FDA approved therapeutic proteins produced in human cells: Idursulfase (Hunter syndrome, approved 2006), Velaglucerase alfa (Type 1 Gaucher disease, approved 2010), rFVIIFc (Hemophilia A, approved in 2014), rFIXFc (Hemophilia B, approved 2014), and Dulaglutide (Type 2 diabetes, approved 2014). The lag in the approval of new products over the past several years, however, suggests that production of therapeutic proteins in human cells remains an infrequently used, niche strategy.

#### 5.3.2 Murine Cells

Expression of FDA-approved therapeutic proteins in murine cells began in the 1990s (Lifely et al., 1995) and continues today despite safety issues including the anaphylaxis-inducing α-Gal epitope (Figure 4B) and high levels of the mildly immunogenic Neu5Gc form of sialic acid (Figure 4A) (Lalonde & Durocher 2017). Although immunogenicity concerns remain for these drugs, safety risks are minimized by pre-screening patients for IgE anti-α-Gal antibodies linked to anaphylaxis, allowing murine-produced mAbs to remain on the market. Mouse myeloma NS0 and Sp2/0 lines are used in biomanufacturing, produceing cetuximab (Erbitux^®^ mentioned earlier) and several mAbs approved up to ∼2015, including palivizumab (Synagis^®^), dinutuximab (Unituxin^®^), necitumumab (Portrazza^®^), and elotuzumab (Empliciti^®^). Similar to human cells, the negligible approval of new products in the past few years suggests that murine cells are unlikely to figure prominently in future biomanufacturing efforts.

#### 5.3.3 Bacteria

During the early development of recombinant DNA technologies in the 1970s, efforts were focused on producing products in bacteria; for example, *Escherichia coli*, was an attractive low-cost, high-yield (e.g., ∼8 g per liter (Menacho-Melgar et al., 2020)) production host. Despite some issues, such as the challenge of purifying recombinant mammalian proteins from bacterial cell components (e.g., the cell wall) and the possibility of endotoxin contaminants (Singh et al., 2016), there were several successfully-produced therapeutic proteins in *E. coli* in the 1980s. These including Humulin^®^ (a recombinant form of insulin), Protropin^®^ and Humatrope^®^ (to treat hGH deficiency), Roferon A^®^ (to treat hairy cell leukemia), and IntronA^®^ (to treat genital warts and hepatitis) (Sanchez-Garcia et al., 2016). In retrospect, the inability of *E. coli* to N-glycosylate proteins, and therefore to take advantage of the concomitant protein folding chaperone system in mammalian cells (Helenius & Aebi 2001), posed a significant challenge during early attempts to express large, difficult-to-fold mammalian proteins in *E. coli* that more often than not resulted in inclusion body formation.

**FIGURE 7:**
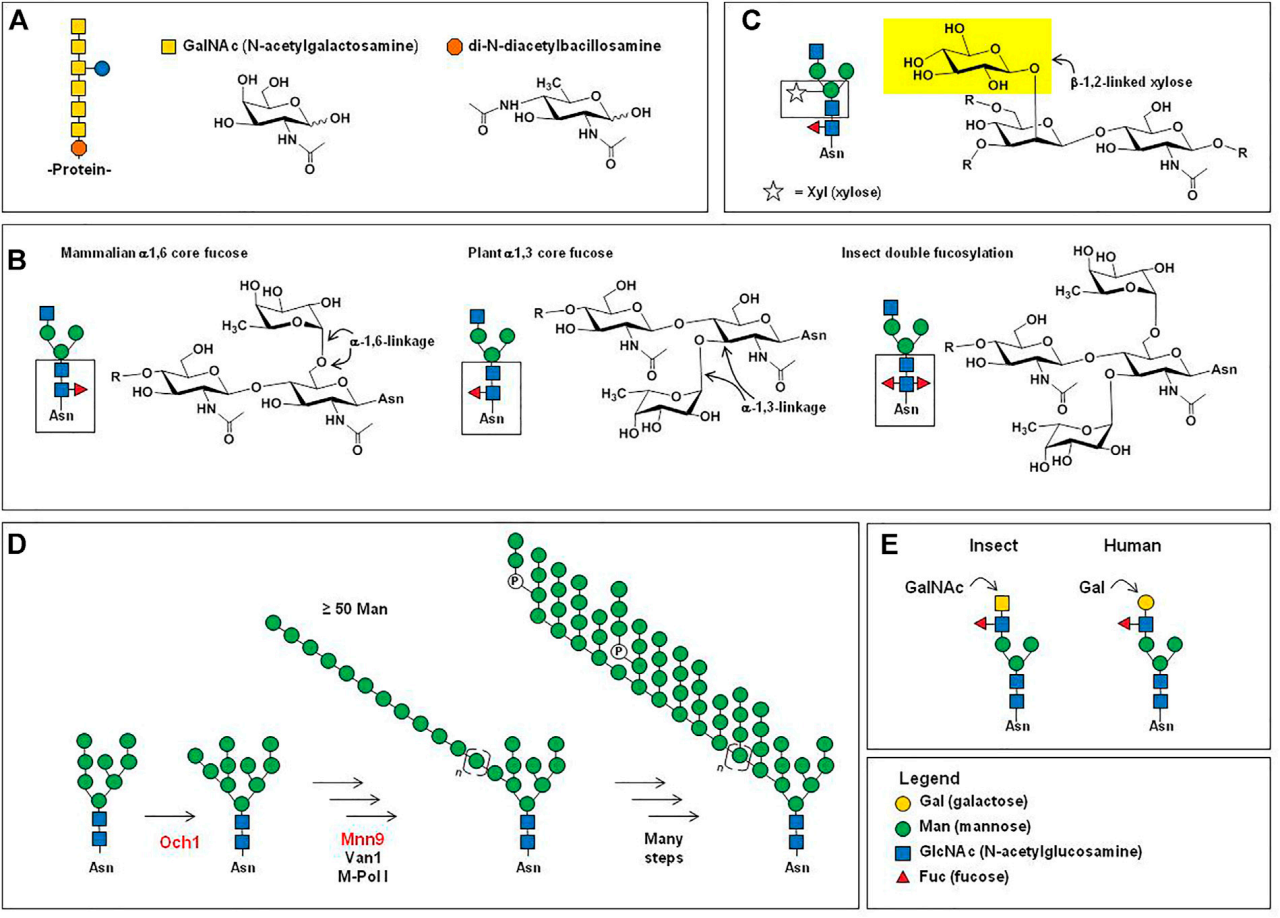
Glycoforms of concern in bacterial, plant, fungal, and insect production systems. **(A)** Efforts to produce glycosylated recombination proteins in bacteria (Section 5.3.3) have resulted in the non-human glycan structure shown. **(B)** Mammalian N-glycans have α1,6-linked core fucose (right), which along with sialic acid, endow IgG antibodies with anti-inflammatory properties; plant cells (Section 5.3.4) produce N-glycan with α1,6-core fucose (center), and insect cells (Section 5.3.6) produce doubly-fucosylated N-glycans (right). **(C)** Xylose, a monosaccharide not present in mammalian N-glycans, is added to plant-produced N-glycans (Section 5.3.4). **(D)** Mannan synthesis in fungi (Section 5.3.5). **(E)** GalNAc incorporation in insects as compared to human galactose addition (Section 5.3.6).

Unlike human and murine cells, which suffer from fundamental limitations for biomanufacturing (e.g., low product yield safety concerns, and high cost), a major detriment of bacterial production systems is their lack of mammalian-type glycosylation. In theory, this pitfall can be overcome, at least in part, by building N-glycosylation capabilities into *E. coli* used for recombinant protein production (Wayman et al., 2019). For example, the protein glycosylation pathway of *Campylobacter jejuni*, a pathogenic bacterium (Szymanski et al., 2002), has been transferred into laboratory strains of *E. coli* (Pandhal & Wright 2010; Wacker et al., 2002). The resulting glycans, however, are distinctly different than human N-glycans (Figure 7A) (Abu-Qarn et al., 2008). For example, they are mainly comprised of GalNAc, a mammalian monosaccharide that does not normally appear in mammalian N-glycans. Similarly, the presence of glucose is unusual for mammalian N-glycans, where this monosaccharide appears in the LLO 14mer precursor structure (Figure 1A) but not in mature N-glycans found on glycoconjugates. Finally, di-N-diacetylbacillosamine is a prokaryotic monosaccharide not found in eukaryotes. Despite technologies to install N-glycosylation pathways in bacteria being in their nascent stages, proof-of-principle experiments (Figure 7A) coupled with continuing robust efforts to improve prokaryote glycosylation (Ding et al., 2019; Wayman et al., 2019; Pratama et al., 2021; Yates et al., 2021) provide hope that in the future, additional therapeutic proteins will be manufactured in bacterial hosts.

#### 5.3.4 Plant Cells

Therapeutic protein production in plants has several advantages, including that the infrastructure for large scale production of crops is in place and in theory requires only water, sunlight, and cheap fertilizers (Burnett & Burnett 2020; Karki et al., 2021). In practice, current plant-based manufacturing uses cell-based methodology rather than field-grown crops but retains advantages over mammalian cell culture. These advantages include a lack of animal products needed for plant cell culture that reduces the risk of viral contamination; the ability to grow cells in inexpensive polyethylene bags rather than stainless steel bioreactors; and room temperature manufacturing without the need for strict temperature control. Counteracting these advantages are the low productivity of plants (e.g., ∼100 mg/kg of plant mass) and the complexity of purifying human proteins from plant matter where cell wall components pose a challenge (Singh et al., 2016; Schillberg et al., 2019).

The use of plant cells for therapeutic protein production is in its infancy, with only one FDA-approved drug. This drug, Elelyso^®^ (i.e., taliglucerase alfa, mentioned above in Section 2.3.3 as a treatment for GD), was approved in 2012 (Fox 2012). Elelyso^®^ illustrates glycosylation differences between plant N-glycans and their mammalian counterparts; for example, the plant ProCellEx^®^ platform directly produces N-glycans with exposed terminal mannose residues (e.g., Glc_2_Man_3_ to Glc_2_Man_5_ structures, Figure 1B) needed for macrophage targeting and uptake (Tekoah et al., 2013). The direct production of MR-targeting glycans in plant cells offers simplicity and cost savings compared to the use of chemical modulators of glycosylation (required for velaglucerase alfa production) or enzymatic modification (imiglucerase) as described in Section 2.3.3.

On a cautionary note, plant cells produce core structures with α1,3-linked fucose not found in humans (human α1,6- vs. plant α1,3-core fucosylation is shown in Figure 7B) and β1,2-linked xylose (Figure 7C) not found in mammalian proteins (Castilho & Steinkellner 2012; Montero-Morales & Steinkellner 2018). Initially, concerns were raised that these non-human glycoforms could be immunogenic in a mildly harmful way reminiscent of Neu5Gc, or possibly with the severe effects of α-Gal. Fortunately, only a small fraction of patients had pre-existing antibodies that recognized these glycans on Elelyso^®^, and those that did experienced no adverse effects (Rup et al., 2017). By contrast, glycan-based immunogenicity of plant-produced blood coagulation factors VIII and XIII remains a substantial impediment to the commercialization of these hemophilia drugs (Top et al., 2019). In some cases, instead of being harmful, the potential immunogenicity of plant glycans has been proposed to enhance cancer vaccines and cancer immunotherapeutics through lectin-based stimulation of antigen-presenting cells (Rosales-Mendoza et al., 2015). Overall, similar to bacterial systems where improved cell hosts are actively being pursued to improve glycosylation, glycoengineering efforts remain underway in plants (Sukenik et al., 2018; Fischer et al., 2021), opening the door for increased use of plants for therapeutic protein production.

#### 5.3.5 Fungi

Several yeast strains, including the widely used production hosts *Saccharomyces cerevisiae* and *Pichia pastoris,* are generally recognized as safe (GRAS) by regulatory agencies. Advantages to fungal production include high yield (up to 12 g/L); cost and safety advantages by avoiding the use of animal products such as FBS during production; and sidestepping the danger of endotoxins from *E. coli* production. A glycosylation-related drawback is the production of hypermannosylated (mannan) N-glycans in yeast that can contain dozens to hundreds of mannose residues [Figure 7D (Orlean 2012)]. These extremely large mannose structures clearly are incompatible with therapeutic glycans. Fortunately, a straightforward solution was found by knocking out two early genes (*Och1* and *Mnn9*, Figure 7D) in mannan biosynthesis [Hamilton & Zha 2015; De Wachter et al., 2021); this approach was commercialized by GlycoFi to humanize yeast glycosylation (Beck et al., 2010)]. The success of such approaches is evident from fungal production systems being second only to CHO cells in the breadth of commercial products; Kulagina and coauthors summarize the use of fungal cells to produce four hormones (Novolin^®^, Glucagen^®^, Valtropin^®^, and Semglee^®^); six vaccines (Recombinvax^®^, Tritanrix-hepB^®^, Gardasil^®^, Mosquirix^®^, Hexacima^®^, and Heplisalv-B^®^); four blood-related proteins (Revasc^®^, Kalbitor^®^, Novothirteen^®^, and Jetrea^®^); one cytokine (Leukine^®^) and one enzyme (Fasturtec^®^) (Kulagine et al., 2021). In addition, glycoengineering strategies are being applied to provide humanized glycan profiles of antibody drugs such as trastuzumab (Herceptin^®^), where fungal Fc domain glycans optimize ADCC (Liu et al., 2018).

#### 5.3.6 Insect Cells

Insect cells, which have been under investigation for recombinant protein production since the 1970s and 1980s (Hollister et al., 1988), represent another low-cost (by using serum-free, chemically defined media), high-yield (∼5 g/L) production system. Manufacturing advantages include no requirement to control CO_2_ levels, relaxed temperature control allowing production at lower temperatures, and reduced biosafety and contamination concerns (Yee et al., 2018). Insect cell lines used include S2 from *Drosophila melanogaster*, Sf9 from *Spodoptera frugiperda,* and High Five^®^ from *Trichoplusia ni* (Yee et al., 2018). Additionally, insect cells can perform both N- and O- glycosylation, they efficiently secrete proteins and can cleave signaling peptides, giving them an advantage over prokaryotic pathways and making them plausible production hosts for large glycoproteins like antibodies (Palmberger et al., 2011; Toth et al., 2014).

There are, however, several glycosylation concerns related to the production of therapeutic proteins in insect cells. For example, insect cells produce simpler N-glycans than mammalian cells, which can affect bioactivity and increase immunogenicity (Geisler et al., 2015; Loos & Steinkellner 2012). Another concern is that although there is some evidence of sialylation in insect cells (Joshi et al., 2001; Kim et al., 2002; Marchal et al., 2001; von Bergen Granell et al., 2011), in general, they do not add this therapeutically important sugar to their gly*c*ans. Another concern is that insect cells have double core fucosylation (Figure 7B), which, if installed in the Fc domain of IgG antibodies likely would impact downstream Fcγ receptor-mediated effector responses. Finally, the presence of GalNAc in the place of galactose on the elaborated antennae of N-glycans produced in insect cells (Figure 7E) is another potential concern. Overall, we note that although there has been decades-long interest in producing IgG antibodies in non-mammalian expression hosts including yeast, plants, and insect cells (Palmberger et al., 2011; Loos & Steinkellner 2012), these efforts have not resulted in commercially successful products. We posit that these difficulties stem in part from glycosylation hurdles, and as such, new glycoengineering approaches will be critical for future production of therapeutic antibodies in a wider range of host cells.

Despite these glycosylation concerns, which have thwarted production of IgG antibodies, several other commercial products have been successfully produced in insect cells. One product is Cervarix^®^, a virus-like particle (VLP) cervical cancer vaccine produced in High Five cells^®^ (Senger et al., 2009). A second is Provenge^®^, the first immunotherapy for hormone-refractory prostate cancer, which is produced in Sf21 cells (Contreras-Gómez et al., 2014). A third is Glybera^®^, a now discontinued adeno-associated virus-based gene therapy for lipoprotein lipase deficiency (LPLD) produced in Sf9 cells (Kurusawa et al., 2020). Finally, Flublok^®^ is a hemagglutinin protein used as an influenza vaccine, which is also made in Sf9 cells (Cox & Hollister 2009). Based on the title of Yee and coauthor’s review article “*The coming age of insect cells for manufacturing and development of protein therapeutics*” (Yee et al., 2018), there is reason for cautious optimism for continued expansion of insect cells as a production platform based on long-standing glycoengineering efforts (Ailor et al., 2000; Hollister et al., 1988; Lawrence et al., 2001; Toth et al., 2014). For example, Mabashi-Asazuma and coworkers have developed a new baculovirus vector that eliminates core α1,3-fucosylation in insect cells (Figure 7B) (Mabashi-Asazuma et al., 2014), decreasing the immunogenicity of glycoproteins produced in these cells.

## 6 Concluding Comments and Future Directions

As the most abundant and varied post-translational modification in mammals in general and humans in particular, glycosylation offers great potential to improve today’s predominant drugs, which are protein therapeutics. As described in this report, which focuses on N-glycans, there are numerous opportunities to glycoengineer current and upcoming proteins to improve their folding, trafficking, ligand interactions, solubility, stability, and to improve the safety, activity, pharmacokinetics, and pharmacodynamics of this increasingly important class of therapeutics. There already are a handful of deliberately glycoengineered products on the market, with prominent examples being afucosylated pro-inflammatory antibodies and β-glucoceraminidase endowed with high mannose-type glycans for macrophage targeting to treat GD. To date, glycoengineered drugs have exploited a single strategy, typically selection of a host cell line capable of biosynthetically producing the desired type of glycosylation. In the future, as already demonstrated pre-clinically by the glycoengineered ENPP1-Fc (Figure 3), multiple glycoengineering strategies (installing new N-glycan sites, production in ST6-overexpressing cells, and media supplementation with a sialic acid precursor) can be productively combined for multifaceted improvement.

Also in the future, additional forms of glycosylation including O-, C-, or S- will provide additional avenues to improve therapeutic proteins. Moreover, the glycoengineering “toolkit” described in Section 5 provides methodology to improve additional biological therapeutics including antimicrobial peptides (AMPs); glycosylated nanoparticles, liposomes, and exosomes for drug delivery and bioimaging; and glycodendrimers (Jain et al., 2012; Grimsey et al., 2020; Torres-Pérez et al., 2020). The safety of these biopharmaceuticals, including toxicity and immunogenicity, are impacted by glycosylation and, similar to antibodies, their glycoprofiles are critical quality control attributes during biomanufacturing (Mastrangeli et al., 2019).

In conclusion, key examples provided for various glycosylation scenarios demonstrate the potential of individualized, targeted glycan modification to improve various therapeutic proteins. As therapeutic proteins advance, the specific adjustment of glycosylation profiles will hold greater importance as biomanufacturers increasingly move from tuning glycosylation to avoid immunogenicity or toxicity to proactively improving drug efficacy.
